# Comparative genomics of *Steinernema* reveals deeply conserved gene regulatory networks

**DOI:** 10.1186/s13059-015-0746-6

**Published:** 2015-09-21

**Authors:** Adler R. Dillman, Marissa Macchietto, Camille F. Porter, Alicia Rogers, Brian Williams, Igor Antoshechkin, Ming-Min Lee, Zane Goodwin, Xiaojun Lu, Edwin E. Lewis, Heidi Goodrich-Blair, S. Patricia Stock, Byron J. Adams, Paul W. Sternberg, Ali Mortazavi

**Affiliations:** Department of Nematology, University of California, Riverside, CA 92521 USA; Department of Developmental and Cell Biology, University of California, Irvine, CA 92697 USA; Department of Biology and Evolutionary Ecology Laboratories, Brigham Young University, Provo, UT 84602 USA; Division of Biology and Biological Engineering, California Institute of Technology, Pasadena, CA 91125 USA; Department of Entomology, University of Arizona, Tucson, AZ 85721 USA; Division of Biology and Biomedical Sciences, Washington University, St Louis, MO 63110 USA; Department of Bacteriology, University of Wisconsin-Madison, Madison, WI 53706 USA; Department of Entomology and Nematology, University of California, Davis, CA 95616 USA; Howard Hughes Medical Institute, Pasadena, CA 91125 USA

## Abstract

**Background:**

Parasitism is a major ecological niche for a variety of nematodes. Multiple nematode lineages have specialized as pathogens, including deadly parasites of insects that are used in biological control. We have sequenced and analyzed the draft genomes and transcriptomes of the entomopathogenic nematode *Steinernema carpocapsae* and four congeners (*S. scapterisci*, *S. monticolum*, *S. feltiae*, and *S. glaseri*).

**Results:**

We used these genomes to establish phylogenetic relationships, explore gene conservation across species, and identify genes uniquely expanded in insect parasites. Protein domain analysis in *Steinernema* revealed a striking expansion of numerous putative parasitism genes, including certain protease and protease inhibitor families, as well as fatty acid- and retinol-binding proteins. Stage-specific gene expression of some of these expanded families further supports the notion that they are involved in insect parasitism by *Steinernema*. We show that sets of novel conserved non-coding regulatory motifs are associated with orthologous genes in *Steinernema* and *Caenorhabditis*.

**Conclusions:**

We have identified a set of expanded gene families that are likely to be involved in parasitism. We have also identified a set of non-coding motifs associated with groups of orthologous genes in *Steinernema* and *Caenorhabditis* involved in neurogenesis and embryonic development that are likely part of conserved protein–DNA relationships shared between these two genera.

**Electronic supplementary material:**

The online version of this article (doi:10.1186/s13059-015-0746-6) contains supplementary material, which is available to authorized users.

## Background

**Fig. 1:**
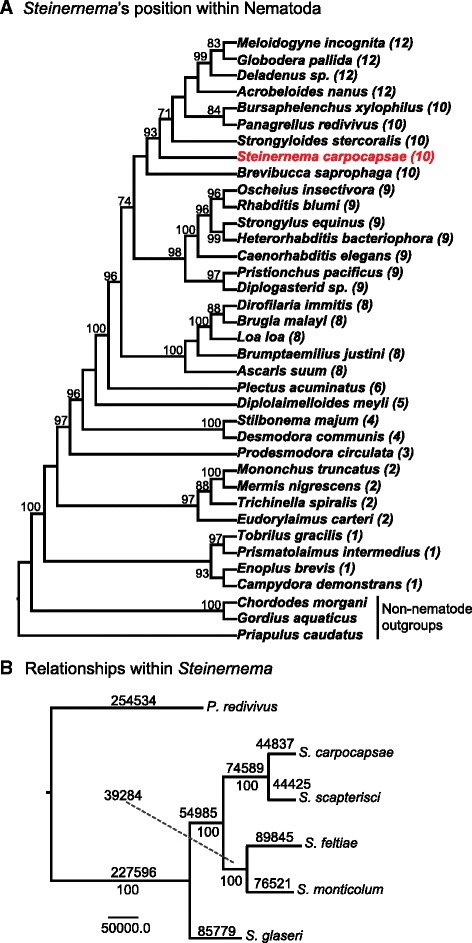
**a** Bayesian analysis of the phylum Nematoda using single locus, partial 18S rDNA sequences. Numbers in parenthesis after scientific names define clade affiliation according to the 12 clade division by Holterman et al*.* [19]. Maximum parsimony bootstrap support values are indicated at the nodes. Values lower than 75 are not reported. **b** Phylogenetic relationships among *Steinernema* species. The maximum parsimony tree is based on a supermatrix of 3,885 strictly homologous genes (1:1 conservation across all species analyzed). The number of changes along each branch is depicted above each branch; bootstrap values (1,000 repetitions) appear at each node

Nematodes are remarkably adept at evolving parasitic lineages with animal-parasitic and plant-parasitic lineages arising many times independently throughout the phylum [1, 2]. To increase our understanding of the evolution of parasitism, we sequenced five species within the insect-parasitic *Steinernema* (Nematoda: Steinernematidae), an intensely studied genus used for decades in biological control against agricultural insect pests and also as a model for animal parasites (Fig. 1a, b, Table 1) [3–5]. Unlike most other sequenced nematodes, which are either harmful or seemingly innocuous to humans, steinernematids are beneficial to humans. *Steinernema* are considered insect pathogenic or entomopathogenic nematodes because of their ability to rapidly (24–48 h) kill an insect host [5–7]. Entomopathogenic lineages have arisen independently at least three times among nematodes [6]. Their ability to kill insects is due in part to their mutualistic association with enterobacteria of the genus *Xenorhabdus*, which are vectored by the only free-living stage in the nematodes’ life cycle, known as the infective juvenile (IJ) (Additional file 1: Figure S1) [5, 8]. Once a suitable host is found, the IJs release the bacteria inside the host, where it grows and helps kill the host by septicemia. The bacteria and host tissues provide a food source for the nematodes to mature and reproduce inside the host cadaver. Once resources are depleted, the bacteria and a new generation of nematodes (IJs) re-establish their association and emerge from the host remains to search for a new host to infect [5, 9]. The bivalent symbiosis (i.e., mutualism and parasitism/pathogenesis) in this tripartite system have made steinernematids (and their bacterial symbionts) an appealing model for understanding mutualism, parasitism, host-seeking, insect immune suppression, and subterfuge [3, 9–11]. In addition to studying parasitism, sequencing five species within a genus (congeners) allowed us to leverage comparative analyses not only within *Steinernema* but among more distantly related taxa such as *Caenorhabditis elegans*. Comparative genomics is a powerful way to understand the complexity of the developmental programs contained within a genome (i.e., promoters, enhancers, transcription-factor binding sites, and the intricate gene regulatory networks that connect transcription factors to each other and their targets [12]). Sequencing closely related organisms for comparative analyses can facilitate the identification of genus-specific gene family expansions and functional non-coding regions of genomes. For example, decoding the developmental programs embedded within the *C. elegans* genome has been challenging, but has benefited from the sequencing of additional congener genomes. The sequencing of the *C. briggsae* genome identified over 1,200 new *C. elegans* genes and helped correct the predicted exon structure for thousands of already annotated genes, but revealed relatively little about conserved non-coding elements [13].

**Table 1:** Features of the *Steinernema* draft genomes

	*S. carpocapsae*	*S. scapterisci*	*S. feltiae*	*S. glaseri*	*S. monticolum*
Estimated genome size (Mb)	85.6	79.4	82.4	92.9	89.3
N50 (bp)	299,566	90,783	47,472	37,444	11,556
N90 (bp)	54,505	15,213	7,098	7,610	2,984
N10 (bp)	979,322	496,671	303,346	112,910	31,326
Number of scaffolds	1,578	2,877	5,839	7,515	14,331
GC content (%)	45.53	47.98	46.99	47.63	42.01
N content (Mb)	2.39	0.76	2.76	3.37	4.34
N content (%)	2.80	0.96	3.36	3.64	4.87
Maximum scaffold size (bp)	1,722,607	1,149,164	1,470,990	339,094	110,081
Number of Augustus-predicted genes	28,313	31,378	33,459	34,143	36,007
Number of Augustus-predicted transcripts	31,944	33,149	36,434	37,120	38,381
Average gene length (bp)	2,030	1,842	1,730	1,855	1,604
Average intron length (bp)	194	153	154	218	161
Average exon length (bp)	212	224	220	216	217
Average intergenic distance (bp)	1,105	723	746	930	761
Average number of exons per gene	5	4	4	4	4
Average number of introns per gene	4	3	3	3	3
GC content in coding regions (%)	51.86	51.92	51.08	53.68	46.92
Number of genes with no introns	4676	5611	6230	7521	6171
Repeat content (%)	7.46	2.75	6.70	5.34	10.49

## Results and discussion

We sequenced and assembled the genomes of five *Steinernema* species (*S. carpocapsae*, *S. feltiae*, *S. glaseri*, *S. monticolum*, *S. scapterisci*) for comparative analysis (Fig. 1, Table 1, Additional file 1: Figure S1). We focused on sequencing *S. carpocapsae* in greater depth than the others to use it as a representative for comparative analyses with other nematode genera. The other species were chosen based on their commercial availability, their evolutionary relationships, and their varied host specificities and foraging strategies. In addition, we sequenced and assembled *de novo* the mRNA of the IJ-stage of each species to aid in genome annotation. Additional RNA was collected for *S. carpocapsae, S. feltiae*, and *C. elegans* at the embryonic, first larval (L1), and young adult stages for a comparative analysis of gene expression, which is discussed below. The final genome assembly sizes ranged from 80 to 90 Mb and 28,000 to 36,000 genes (Fig. 1, Table 1, Additional file 1: Figure S2, Additional file 2: Table S1) and *S. carpocapsae* was the best assembled genome (scaffold N50 = 299 kb) with an estimated genome completeness of 98 % (Fig. 1, Additional file 2: Table S2). Detailed methods for assembly and annotation can be found in the “Methods” section.

### Phylogenetic analysis

Although taxon selection clearly influences phylogenomic accuracy [14], sequencing the genomes of multiple species in the same genus should increase confidence in our ability to recover their evolutionary history [15–18]. Current best estimates place *Steinernema* in Holterman clade 10 and thus closely related to the sequenced nematode *Bursaphelenchus xylophilus*, a plant-parasite, and the free-living *Panagrellus redivivus* (Fig. 1a) [1, 2, 19]. Previous attempts to recover relationships among different species of *Steinernema* resulted in several poorly resolved/supported nodes, likely due to the limited number of molecular markers used and their homoplastic and/or plesiomorphic nature [20]. We evaluated the relationships among the five *Steinernema* using a supermatrix of 3,885 strictly orthologous genes (1:1:1:1:1:1), with *P. redivivus* as our out-group taxon (Fig. 1b). The relationships we recovered are strongly supported but differ from previous hypotheses in that *S. monticolum*, which was chosen for sequencing based on its hypothesized close relationship to *S. carpocapsae* and *S. scapterisci* [16], was more closely related to *S. feltiae* than any of the other nematodes in our analysis.

### Gene orthology

**Fig. 2:**
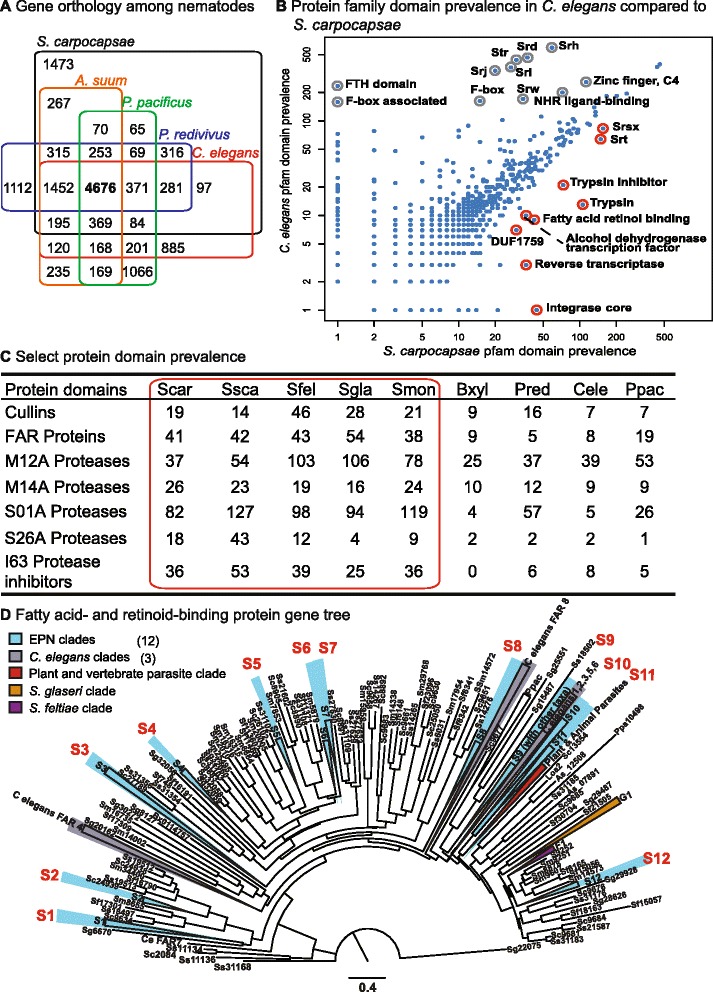
**a** Gene orthology clusters among five sequenced species of nematodes, with 4,676 orthologous clusters being shared among all five species and 1,473 clusters being unique to *S. carpocapsae*. **b** The abundance of Pfam protein family domains in the *C. elegans* and *S. carpocapsae* genomes. The nine most enriched Pfam domains (biggest absolute difference) in *S. carpocapsae* relative to *C. elegans* are highlighted in *red* while the eleven most enriched Pfam domains in *C. elegans* relative to *S. carpocapsae* are highlighted in *gray*. **c** Select Pfam domains that are enriched in the sequenced steinernematids compared to other nematode species. **d** Protein neighbor-joining tree of the fatty acid- and retinol-binding proteins in nematodes. Monophyletic protein clades with at least one protein from each of the five *Steinernema* spp. are highlighted in *blue*. This figure illustrates both the abundance and diversity of FAR proteins among steinernematids. *EPN* entomopathogenic nematodes, *FAR* fatty acid- and retinol-binding proteins

The predicted proteome of an organism can highlight the conserved proteins shared with other species in its phylum and genus as well as the specializations that allow each species to adapt to its environment. The predicted proteome of 28,313 *S. carpocapsae* proteins was compared to the predicted proteomes of eight other nematode species and an insect out-group: *P. redivivus*, *C. elegans*, *Pristionchus pacificus*, *Meloidogyne hapla*, *Bursaphelenchus xylophilus*, *Brugia malayi*, *Ascaris suum*, *Trichinella spiralis*, and the parasitoid wasp *Nasonia vitripennis* (Fig. 2a) [21–29]. The other nematodes used in this comparison included free-living (*C. elegans* and *P. redivivus)*, necromenic (*P. pacificus*), plant-parasitic (*M. hapla* and *B. xylophilus*), and vertebrate-parasitic species (*B. malayi*, *A. suum*, and *T. spiralis*) (Fig. 1a). Most of the predicted *S. carpocapsae* genes had homologs (BLASTp e-value cut-off: 10^-5^) in one or more species included in this analysis; 10,350 orthology clusters included 17,653 (62.3 %) *S. carpocapsae* proteins. A total of 266 of these clusters were found exclusively in nematodes. We found that 1,633 orthology clusters included at least one protein from each of the ten taxa analyzed, 486 of which were strictly conserved at a 1:1 ratio across all taxa (Additional file 3). While most molecular phylogenetic studies of nematodes rely on one or a few genes, this set of 486 highly conserved genes is a source of characters that could increase phylogenetic resolution in future analyses [2, 19]. In this analysis, there were 10,660 orphan *S. carpocapsae* proteins (37.7 % of the proteome) that did not cluster with any other proteins in the dataset, suggesting either that they are uniquely derived within *S. carpocapsae,* or that they have evolved such disparate primary sequences that they cannot be linked to their orthologs by sequence similarity alone. Protein orthology was also evaluated using the predicted protein sets for the five steinernematids sequenced and included either *C. elegans* or *P. redivivus* as out-group taxa. In these analyses the number of *S. carpocapsae* orphan proteins changed little, from 37.7 % in the phylum-wide analysis to 32.3 % or 32.4 % respectively (Fig. 2, Additional file 2: Table S1). Of the predicted *S. carpocapsae* genes, 80.5 % had at least partial RNA-seq transcript support (Additional file 2: Table S3). It is remarkable that these putative orphan proteins consistently included more than 30 % of the predicted proteome even when examining species within *Steinernema,* whereas a detailed genomic analysis of 12 species of *Drosophila* revealed the percentage of orphan proteins ranges from 14 % to 27 % in that genus [30].

### Protein family domain abundance

We then analyzed the predicted protein domains to understand which gene families have undergone expansions in *Steinernema* that may have contributed to adaptation to a parasitic lifestyle. The *S. carpocapsae* proteome was predicted to have a total of 17,518 Pfam domains from 3,256 different Pfam accession categories. The relative Pfam domain abundances of the *S. carpocapsae* genome were compared to those in the parasitic *Bursaphelenchus xylophilus*, *Brugia malayi*, and *Ascaris suum*, as well as the free-living *C. elegans* (Fig. 2b, Additional file 1: Figures S3–S6). Overall, most Pfam domains were detected at similar levels in both genomes, with some notable exceptions. For example, while most transcription factor domains showed similar prevalence in both genomes, we found the expected enrichment of C4 zinc fingers in *C. elegans* that are associated with nuclear hormone receptors, as well as a novel three-fold enrichment of the alcohol dehydrogenase transcription factor Myb/SANT-like domain in *S. carpocapsae* (Fig. 2b). The *S. carpocapsae* genome appears to be enriched in proteases, protease inhibitors, certain families of G protein-coupled receptors (GPCRs) , and fatty acid- and retinol-binding (FAR) proteins, among others (Fig. 2b, Additional file 1: Figures S3–S6). The abundance of predicted Pfam domains from *S. carpocapsae* was compared with the other four *Steinernema* species we sequenced (Fig. 2, Additional file 1: Figures S7–S10). The domain richness of certain types of proteases, protease inhibitors, and certain families of GPCRs varied widely between the different species of *Steinernema*, though some enrichments were shared, such as the greater abundance of certain protease and protease inhibitor families, and FAR proteins, which appeared in all *Steinernema* species and are discussed separately below (Fig. 2c).

### Putative parasitism genes: proteases and protease inhibitors

Proteases (peptidases) are involved in a wide variety of biological functions including development, digestion, signal transduction, and immune responses [31]. Of particular relevance in these genomic analyses is the role proteases play in parasitism, such as tissue penetration and immune suppression or evasion [32–35]. A total of 654 peptidases were identified in the *S. carpocapsae* genome (Fig. 2, Additional file 2: Table S4). These can be broken down into five key classes based on the chemical groups that function in catalysis: aspartic (6.3 %), cysteine (19 %), metallo- (32.7 %), serine (37.6 %), and threonine (4.1 %). Because steinernematids can be lethal even without their pathogenic symbionts [36–39] and proteolytic activity is higher in the excreted-secreted products in more virulent strains [39, 40], proteases were among the first products examined in relation to the lethality of *Steinernema* nematodes and have been suggested to be actively pumped into host tissues by parasitic nematodes [33, 35, 41–43]. Steinernematids have more predicted proteases (Fig. 2, Additional file 2: Table S4) than any other nematode sequenced to date. This correlates with the remarkably broad host ranges of many *Steinernema* species, which can infect multiple species across many insect orders in some cases, whereas other parasitic nematodes have more restricted or specialized host ranges. Breaking the proteases into subclasses highlights species-specific expansions of serine and metalloproteases among *Steinernema* species. However, the abundance of aspartic, cysteine, and threonine proteases is relatively similar across nematodes (Fig. 2c, Additional file 2: Tables S4–S7). The serine and metalloproteases are the most highly represented families in nematode excreted-secreted products, suggesting that they play a role in parasitism [42]. We found *Steinernema*-specific expansions of chymotrypsin-like (S01A), Lon-A-like (S16), and signal peptidase I-like (S26A) serine proteases and expansions of the astacin (M12A), carboxypeptidase A1-like (M14A), and the pitrilysin (M16A) metalloproteases. Whereas chymotrypsin-like and carboxypeptidase A1-like proteases were expanded in all five of the *Steinernema* spp*.* when compared to other nematodes, other proteases such as the Lon-A-like, signal peptidase I-like, astacin, and pitrilysin proteases were only expanded in certain species (Fig. 2c, Additional file 2: Tables S8–S11). These expansions represent putative parasitism genes and may affect the host-range and specificity of these species, influencing their ability to infect and avoid the immune response of certain potential host species. Some proteases in these expanded families have characterized roles in parasitism in *Steinernema*. For example, an S01A chymotrypsin-like protease from *S. carpocapsae* has increased expression in IJs exposed to waxworm hemolymph and suppresses waxworm prophenoloxidase activity and immune encapsulation in vitro [33]. Additional biochemical and molecular studies are needed to understand immune suppression and evasion by steinernematids and the role proteases play in these processes.

Previous work has shown a functional role for several proteases in the parasitism of insects by entomopathogenic nematodes. For example, Toubarro et al*.* identified an *S. carpocapsae* serine protease that hydrolyzes extracellular matrix proteins and induces apoptosis of insect cells [35]. Two other *S. carpocapsae* serine proteases are involved in immune subversion by inhibiting insect prophenoloxidase activity and disrupting cellular encapsulation by the insect immune response [33, 44]. Also, an *S. carpocapsae* astacin is upregulated in IJs upon infection of an insect host, suggesting a role in the infection process [45]. Our findings further support the notion that certain families of proteases play a role in parasitism and may have shaped niche partitioning among the many species of insect parasites.

The virulence of parasitic nematodes is heavily influenced by not only proteases but also protease inhibitors [43, 46]. In addition to the expansion of proteases, the *Steinernema* genomes show large expansions of several specific protease inhibitor families, such as the I4 serine protease inhibitor (serpin) family, the I8 chymotrypsin/elastase inhibitor family, and the I63 pappalysin-1 inhibitor family (Fig. 2c, Additional file 2: Table S12) [47]. This genus-specific expansion in *Steinernema* species and the known role of many protease inhibitors in parasitism, particularly serpins (reviewed by Molehin *et al.* [48]), suggests that these protease inhibitors are putative parasitism genes likely used by steinernematids to successfully infect hosts. We examine stage-specific gene expression of some of these putative parasitism genes below (S26 proteases and I63 protease inhibitors). Future investigations of the expression context and biochemical function of the expanded proteases and protease inhibitors identified here in these parasitic nematodes might reveal that they facilitate the parasitism of insects and that the various expansions and retractions of these families among steinernematids influence host range and specificity.

### Putative parasitism genes: fatty acid- and retinol-binding proteins

The fatty acid- and retinol-binding protein (FAR) gene family represents another dramatic case of genus-wide expansion in *Steinernema* (Fig. 2d, Additional file 1: Figure S11, Additional file 2: Table S13). *Steinernema* species have between 38 and 54 FAR proteins compared to 19 in *P. pacificus* and fewer in the other nematodes we examined (Additional file 1: Figure S11, Additional file 2: Table S13). FAR proteins are a family of lipid-binding proteins that have high binding affinities for fatty acids, retinol, and retinoic acid and are unique to nematodes [49]. They are important in the growth, development, and reproduction of *C. elegans*, which, like most if not all nematodes, is auxotrophic for sterols. However, FAR proteins were originally discovered in vertebrate-parasitic nematodes, where, in addition to their role in growth and development, they are thought to play a key role in parasitism by functioning in the sequestration of host retinoids as well as by contributing to immune evasion or suppression, though their exact role is not understood [49, 50]. Although parasitism arose independently multiple times among nematodes, FAR proteins have been implicated in the parasitism of plants, invertebrates, and vertebrates across all of the parasitic lineages, suggesting that this protein family is particularly important to parasitism (Fig. 1b) [49, 51, 52]. We examine the stage-specific expression of FARs and explore their genome architecture below. While the function of these proteins in parasitism remains to be shown, one possibility is that they interact with eicosanoids—fatty acids involved in immunological signaling in plants, mammals, and insects [53–55]. Inhibiting eicosanoid biosynthesis has been shown to reduce the melanotic encapsulation response of insects, which is thought to be insects’ primary defense against nematode parasites [10, 56]. For example, *Xenorhabdus nematophila*, the insect-pathogenic symbiont of *S. carpocapsae*, has been shown to dampen the host insect immune response by inhibiting eicosanoid synthesis in infected insects, increasing the likelihood of a successful infection by *S. carpocapsae* [57, 58]. Thus inhibiting eicosanoid biosynthesis in hosts is one way that parasitic nematodes may suppress host immunity.

### Differential gene expression analysis

**Fig. 3:**
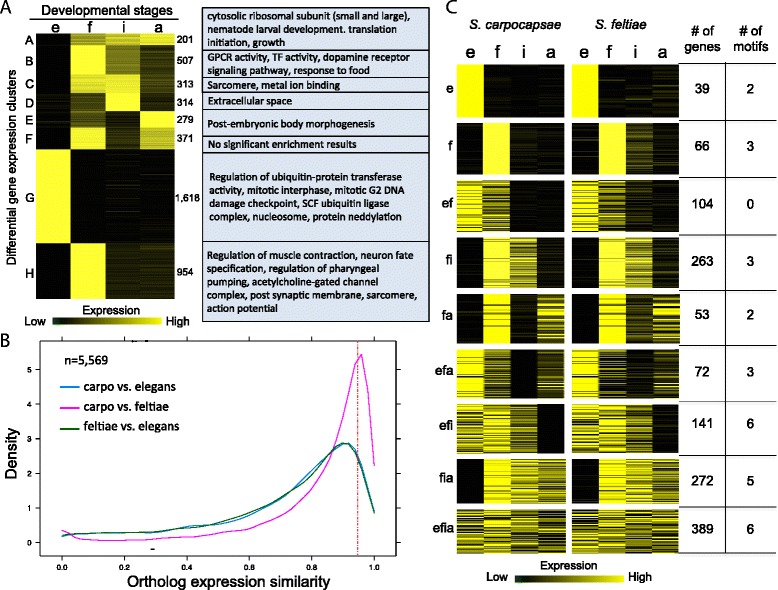
**a** Heat map of 4,557 differentially expressed (DE) genes (FDR < 1 × 10^−5^, fold change > 4×) during *S. carpocapsae* development. Gene Ontology term enrichment analysis was performed on the DE gene sets with Blast2GO (Fisher’s exact test, FDR < 0.01). Gene expression for each stage for each gene was scaled so that the total expression of the row sums to 1. **b** Plot showing the distribution of gene expression profile similarities for 5,569 1:1:1 orthologs between species pairs during development. Ortholog expression (TPM) during development for each species was treated as a vector, and ortholog expression similarity was determined by calculating the cosine similarity of the vectors, where 1 corresponds to identical expression profiles, and 0 corresponds to divergent expression profiles. Orthologs with conserved stage-specific expression profiles have similarity measures > 0.95. **c** Heat map showing the ortholog expression profiles of the conserved stage-specific orthologs (cosine similarity > 0.95) in (b) in *S. carpocapsae* and *S. feltiae*. Gene expression is scaled so that the total expression across a row sums to 1 as in (a). The number of genes in each gene set and the number of significant non-redundant motifs that were derived from each gene set are shown to the *right. e* embryonic, *f* first larval, *i* infective juvenile, *a* adult developmental stages

**Fig. 4:**
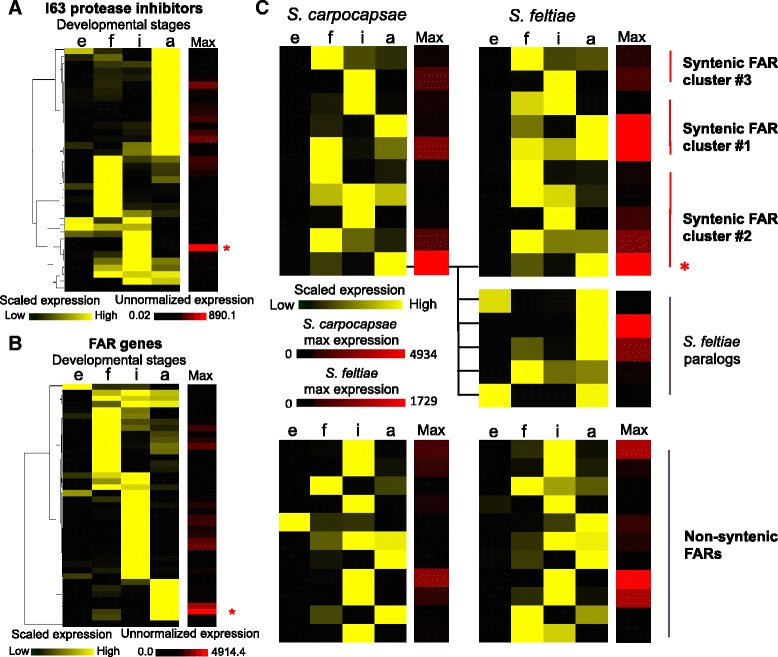
Stage-specific gene expression of FAR proteins and protease inhibitors in *S. carpocapsae*. Heat map of the scaled gene expression of **a** 37 I63 protease inhibitors, and **b** 41 FAR proteins in *S. carpocapsae*. Expression of each gene was scaled between 0 (minimum expression, *black*) and 1 (maximum expression, *yellow*) across the developmental stages, and hierarchically clustered based on the expression profiles. The *Max* column shows each gene’s maximum non-normalized expression value (FPKM) that was achieved during the time course. Genes that have high expression are in *red*, while genes that have low expression are in *black*. The *asterisk* indicates the gene that has the highest expression. **c** Gene expression of 22 1:1 orthologous FAR genes across three syntenic FAR clusters and the non-syntenic FARs were compared between *S. carpocapsae* and *S. feltiae*. For each FAR ortholog, gene expression was scaled between 0 (minimum expression) and 1 (maximum expression) across the developmental stages. The *Max* column shows the maximum gene expression levels of the FARs in *S. carpocapsae* and *S. feltiae*. Five *S. feltiae* paralogs of the bottom-most ortholog in FAR syntenic cluster #2 are shown separately beneath the heat map. *e* embryonic, *f* first larval, *i* infective juvenile, *a* adult developmental stages

We collected mRNA from the early embryonic, L1, IJ, and young adult stages of *S. carpocapsae* in biological replicates for differential expression analysis. A total of 4,557 genes were differentially expressed (DE) in *S. carpocapsae* across the time course [false discovery rate (FDR) < 1×10^−5^ and fold changes > 4×] (Fig. 3a, Additional file 2: Table S14, Additional file 4)*.* Gene Ontology (GO) analysis of the DE stage-specific gene sets revealed enrichment for mitosis-related GO terms (1,618 genes) in the early embryonic stage. This agrees with what has been observed in *C. elegans*, for which the majority of cell divisions occur during the first half of embryogenesis [59]. DE L1 genes (954 genes) were enriched for GO terms involved in feeding and sensation, neuronal cell fate, and muscle contraction. While muscle contraction should be important for all post-embryonic stages, these particular functions might be overrepresented in the L1 stage because the cells that carry out these functions make up a greater proportion of the body mass of the organism at this stage relative to other stages. DE genes in all the post-embryonic developmental stages were associated with ribosomal constituents, translation, and growth (201 genes), reflecting the dependence of early embryos on maternal ribosomes and other translation machinery. Moreover, while cellular division occurs primarily during embryonic development and during portions of larval stages [60], cellular growth of particular cell types occurs primarily over the duration of each developmental stage. These results show that our stage-specific gene sets capture the biologically meaningful processes occurring during these developmental stages and likely reflect processes essential for *S. carpocapsae* development. We also investigated the similarity of transcript isoform expression during development in *S. carpocapsae* and *S. feltiae* and found that a large fraction of isoform pairs, 1,377 out of 3,202 (43 %) in *S. carpocapsae* (Additional file 5), and 1,189 out of 2,333 pairs (51 %) in *S. feltiae* have diverged in their expression during development (Additional file 1: Figure S12). We further used our data to examine the stage-specific expression of the FAR proteins, the S26 proteases, and the I63 protease inhibitor family (Fig. 4, Additional file 1: Figure S13). In each of these protein families, a single ortholog was very highly expressed and dominated the other orthologs, and many of the orthologs had distinct stage-specific expression in the two species. We identified DE genes in each of these categories in different *S. carpocapsae* developmental stages (Fig. 4a, b), suggesting further specializations in parasitism.

### Gene expression conservation across species

The expression of orthologous genes during development is known to diverge [61, 62]. In order to identify genes with conserved patterns of stage-specific expression across closely and more distantly related species, mRNA from the corresponding embryonic, L1, IJ, and adult stages of *S. feltiae* and *C. elegans* was collected and sequenced for comparison to *S. carpocapsae* (Fig. 3, Additional file 2: Table S14). We limited our analysis to 5,569 1:1:1 orthologs present in all three species to avoid the complications of divergent expression due to gene duplications. We used two methods for determining conserved stage-specific ortholog expression. The first method binarized stage gene expression values using a flexible threshold to sort genes into stage-specific sets. We used this method to quantify the number of orthologs that are “on” and “off” in the same developmental stages between species. We found that 79.3 % (4,416/5,569) of these orthologs had conserved expression between *S. carpocapsae* and *S. feltiae*, whereas pairwise comparisons of the expression of each *Steinernema* species to *C. elegans* showed lower overall conservation of stage-specific expression of 61–63 % (3,504/5,569 and 3,432/5,569) (Additional file 1: Figure S14). Nevertheless, given that the steinernematids are phylogenetically distant from *C. elegans* yet share expression of more than two-thirds of their 1:1:1 orthologous genes, these results suggest that gene expression of this core set of unduplicated genes is highly conserved among nematodes. In a separate analysis, we treated the expression of each ortholog during development in each species as a vector and calculated their cosine similarities to address whether the ortholog expression profiles parallel each other during development. We found that 1,441 out of 5,569 orthologs (25.8 %) had a conserved pattern of stage-specific expression (ortholog expression similarity > 0.95) between *S. carpocapsae* and *S. feltiae* (Fig. 3b, Additional file 6), whereas there was more divergence with *C. elegans*. Only 541 (9.7 %) orthologs were conserved in stage-specific expression between *C. elegans* and *S. carpocapsae* and 490 (8.7 %) between *C. elegans* and *S. feltiae* when all developmental stages were considered.

Using the stage-specific gene expression data, we determined the gene expression levels of 41 FARs, as well as the expression levels of a family of serine proteases and protease inhibitors (S26 and I63) that may play a role in the parasitic lifestyle of *S. carpocapsae*. We found that sets of the I63 protease inhibitors were expressed at particular post-embryonic developmental stages, with the highest expression levels occurring in the IJ (839.8 FPKM) and adult stage (239.4 FPKM) (Fig. 4a). These are the stages most important in the successful infection of an insect host and these expression data support the notion that I63 protease inhibitors are important for *S. carpocapsae* parasitism. However, most of the S26 proteases (14/17 proteases) were expressed primarily in the embryonic stage, suggesting that they are involved in development rather than the parasitism of insects by *Steinernema* (Additional file 1: Figure S13). Additionally, we found that 39 of 41 FAR genes were primarily expressed during the post-embryonic stages (Fig. 4b), and that about half of these genes appeared in clusters in the genome sequence. Of the eight *C. elegans* FAR genes, only *far-*1 was conserved in the steinernematids. This gene is reported as having highest expression in L3 *C. elegans* worms [63]. We confirmed this, seeing high expression in the dauer and L1 stages (Additional file 1: Figure S15). Among the stages we tested, *Steinernema far-1* orthologs had highest expression in L1 (Additional file 1: Figure S16), suggesting that they function in development and not parasitism, but this remains to be tested.

### Genome conservation and synteny analysis

**Fig. 5:**
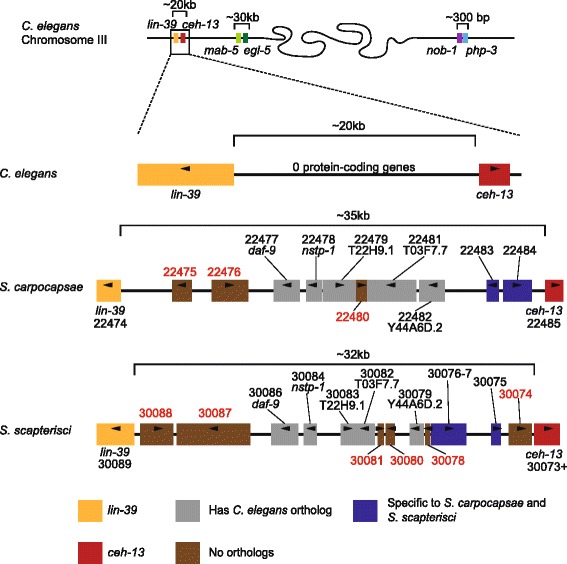
Hox cluster architecture in *Steinernema.* Comparisons of the Hox clusters of *C. elegans, S. carpocapsae*, and *S. scapterisci*. Each cluster is mapped at the same scale, with the *colored boxes* representing different putative genes between the *lin-39* and *ceh-13* orthologs. Genes marked in *blue* are specific to *Steinernema*, not having orthologs in *C. elegans. Gray* genes have a *C. elegans* ortholog, though they are not syntenic in the nematodes compared. Genes marked in *brown* are unique, not having obvious orthologs in the other nematodes in this comparison

The evolution and conservation of non-coding regions and their relationship to gene expression remains an open problem, with the central premise of comparative genomics being that conservation is one signature of potential function and functional linkage of elements with genes. We therefore aligned the sequences of the five *Steinernema* genomes globally to find such linkages and to reveal patterns of evolution in syntenic gene clusters. A genome-wide analysis of syntenic 1:1 orthologs of *S. carpocapsae* with each of the four congeners we sequenced revealed that the most closely related species pair, *S. carpocapsae* and *S. scapterisci,* had the most syntenic 1:1 orthologs, with 11,272 of 12,395 (90.9 %) 1:1 orthologs in synteny in scaffolds containing at least two syntenic orthologs, and 6,576 of 12,395 (53.0 %) 1:1 orthologs in synteny in scaffolds with ten or more syntenic 1:1 orthologs (Additional file 2: Table S15). However, the greatest stretch of syntenic 1:1 orthologs was between *S. carpocapsae* and *S. feltiae*, with 191 orthologous genes spanning a distance of 878 kb in *S. carpocapsae* and 794 kb in *S. feltiae*, which is a rather unexpected finding given the better assembly of *S. scapterisci* (scaffold N50 = 90,783 bp) compared to *S. feltiae* (scaffold N50 = 47,472 bp) (Table 1, Additional file 2: Tables S15 and S16). A local analysis of synteny was done to investigate two noteworthy sets of genes. The first set of genes was the nematode Hox cluster that is quickly evolving in all nematodes [64, 65]. All of the core nematode Hox genes (*ceh-13*, *lin-39*, *mab-5*, *egl-5)* were found in most of the *Steinernema* assemblies (Fig. 5, Additional file 1: Figure S17A), and an expansion was identified in the anterior portion (*ceh-13*, *lin-39*) of the Hox cluster, where the gap between *ceh-13* and *lin-39* is 19 kb in *C. elegans* and has expanded to 35–43 kb in several of the *Steinernema* genomes considered in this study (Fig. 5, Additional file 1: Figure S17A,B). Also, approximately 15 expressed genes have become embedded between *ceh-13* and *lin-39* in *Steinernema* genomes*,* the 1:1 orthologs of which are not present anywhere near the Hox genes or each other in *C. elegans*, suggesting that the distance between Hox genes in the cluster in *Steinernema* is in the process of expanding (Fig. 5, Additional file 1: Figure S17C–E).

The second set of genes we investigated was the family of FAR genes in *Steinernema*. We found a total of 22 out of 41 FAR genes in synteny across three distinct syntenic clusters in *S. carpocapsae*. By examining the location of the 1:1 orthologs of these genes in the other *Steinernema* species, we found that the majority of these orthologs are also syntenic in *S. scapterisci* (13/17 1:1 orthologs) and *S. feltiae* (10/12 1:1 orthologs) and that their expression during development is also conserved across the *Steinernema* species (Additional file 1: Figure S18). Interestingly, we also saw that the most highly expressed FAR in *S. carpocapsae* has five paralogs in *S. feltiae*, with one dramatically changing its expression pattern from adult to embryonic stage, which suggests that this family is undergoing further rapid functional evolution within *Steinernema* (Fig. 4c).

### Conserved non-coding networks

**Fig. 6:**
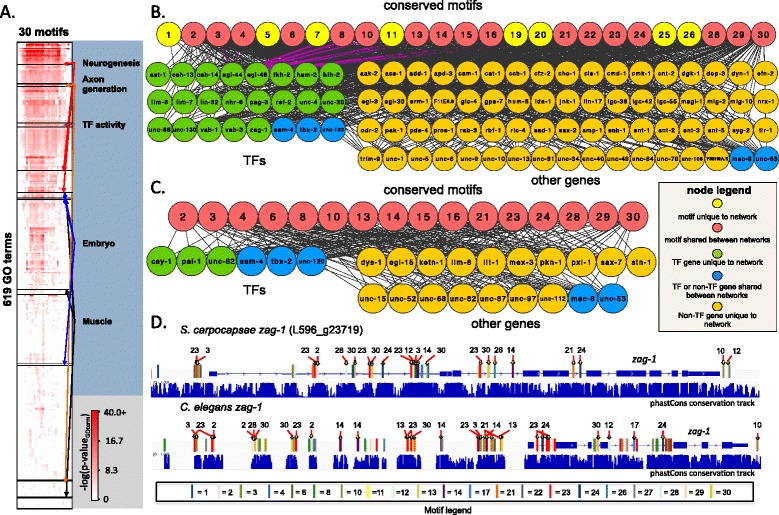
Conserved non-coding networks in *Steinernema* and *Caenorhabditis.* **a** A hierarchically clustered heat map of 30 derived regulatory motifs and the GO terms that the target genes of these motifs are enriched in. Only motif-GO term associations that are shared between *S. carpocapsae* and *C. elegans* are shown. *p*-values depicted are from *C. elegans* associations. *Colored arrows* point to single GO term or groups of GO terms that belong to the four developmental categories shown. **b** A network of conserved *S. carpocapsae* and *C. elegans* motif-target gene associations related to neurogenesis GO terms. Only nodes for motifs and downstream genes with degrees ≥5 are shown in the network. **c** A network of conserved *S. carpocapsae* and *C. elegans* motif-target gene associations related to muscle GO terms. Only nodes for motifs and downstream genes with degrees ≥5 are shown in the network. **d** *zag-1* gene model in *S. carpocapsae* and *C. elegans* showing conserved motifs, and well as conserved regulatory modules (clusters of conserved motifs). Sequence conservation tracks are displayed below each gene model. Associations between *zag-1* and motifs are highlighted in *red* in the neurogenesis network. *GO* Gene Ontology

Non-coding cis-regulatory elements bound by transcription factors control the expression of their associated genes; two of the major goals of comparative genomics are the discovery of these elements and of the gene regulatory networks encoded by these tshared elements. We expect that genes with conserved gene expression profiles would share conserved cis-regulatory elements. Several studies of the evolution of gene expression have shown that cis-regulatory changes represent a major component (reviewed in [66]). In addition, rapid “re-wiring” of gene regulatory networks due to site turnover even between relatively closely related species in mammals and flies makes it difficult to find these cis-regulatory elements using global alignments alone (reviewed in [67]). Our previous experience with the small amount of non-coding sequence alignment between two distantly related species within the same genus, *C. elegans* and *C. angaria*, suggested that we would find very little directly alignable non-coding sequence between two distant genera [68]. We therefore postulated that, while the sets of orthologs conserved in stage-specific gene expression during *Steinernema* and *Caenorhabditis* development (Fig. 3b) are likely to be regulated by shared sets of non-coding, cis-regulatory elements, we would need to use a strategy that leverages non-coding alignability within a genus but does not require it for comparison with orthologs in a more distant genus such as *Caenorhabditis*. We filtered any conserved sequences that overlapped either gene models or transcripts assembled from our RNA-seq data in *S. carpocapsae* (Additional file 7). We found that 14.8 Mb (17.2 %) of the *S. carpocapsae* genome comprises conserved coding sequence while a further 4.5 Mb (5.2 %) comprises conserved non-coding sequence (Additional file 1: Figure S19A). We then searched for novel regulatory motifs around nine sets of *Steinernema* orthologs with conserved expression patterns between *S. carpocapsae* and *S. feltiae* (Fig. 3c, Additional file 1: Figure S19B), and found 30 non-redundant motifs (Additional file 2: Table S17, Additional file 8), 24 of which matched the sequences of one of more motifs from the WormBase database (*p*-value < 1e^−4^ and e-value < 0.5) (Additional file 2: Table S18). All 30 of these motifs were mapped to the conserved non-coding regions in *S. carpocapsae* and *C. elegans* (from multiple sequence alignment of seven *Caenorhabditis* species, UCSC), revealing that they are enriched in the neighborhood of genes involved in the same biological processes (GO terms) (Fig. 6a, Additional files 9, 10). We found that the shared enriched GO terms that also involved a high percentage of 1:1 orthologs between the two species were related to processes such as neurogenesis, axonogenesis, embryogenesis, and muscle development. We further restricted ourselves to orthologous genes in *S. carpocapsae* and *C. elegans* that shared the same motifs and built three representative subnetworks of motifs-to-genes based on these GO enrichments (Additional file 2: Table S19). These networks revealed conserved associations between regulatory motifs and their target genes between the two species for genes in the core of neurogenesis/axonogenesis, embryogenesis, and muscle development (Fig. 6a–c, Additional file 1: Figure S20). In particular, 25 regulatory motifs (degree ≥ 5) potentially regulate 92 neurogenesis genes whereas 16 overlapping regulatory motifs regulate 25 muscle development-related target genes in both *C. elegans* and *S. carpocapsae* (Fig. 6b, c, Additional files 11, 12, and 13). In order to verify that the motif-associated GO term enrichments we obtained were not due to chance, we created 100 randomized GO term sets by shuffling all of the annotated *S. carpocapsae* gene GO terms. We reassigned the GO term sets to new genes, and ran all 30 motif-associated gene sets through a Fisher’s exact test using these randomized GO sets (30 motifs × 100 randomizations = 3,000 Fisher’s exact tests in total). We were unable to recover GO term enrichments for any of the GO terms that comprised the neuronal, embryo, or muscle networks for any of our motifs using randomized shuffling (0/3,000, FDR < 0.05), suggesting that the GO enrichments we identified are meaningful.

Multiple motifs from the same networks clustered together in or near some of the orthologous target genes of *S. carpocapsae* and *C. elegans*. Some of these motif clusters showed conserved order and position, whereas others showed variation in order only, position only, or variation in both between species (Fig. 6d, Additional file 1: Figure S21). Comparative analysis of the *Steinernema* congeners led to the identification of these conserved motifs. We found them conserved in *C. elegans* and enriched near genes influencing similar biological processes in a distantly related genus. This finding suggests that they are under evolutionary selection, although their functionality remains to be tested.

## Conclusion

The sequencing of multiple species of *Steinernema* enabled us to identify gene family expansions that are consistent with and likely important to the particular biology of these species as parasites; to generate new hypotheses about genes likely to be important in parasitism; to explore their genealogy more deeply than ever before and refine our understanding of their relationships to each other as well as define other phylogenetic markers that could be used in subsequent analyses; to identify stage-specific enrichment of functional gene classes; to demonstrate that the differential expression of stage-specific genes is influenced by phylogeny; to explore the evolution of the developmental control genes in the Hox gene cluster and diagnose expansion and rapid evolution of this cluster; and to identify previously unknown conserved non-coding regulatory motifs that regulate similar biological processes in distantly related organisms [69]. Our results point to a core set of conserved motifs, functioning in both *C. elegans* and *S. carpocapsae,* that regulate similar biological processes key to proper nematode development across vast phylogenetic distance. These motifs are not detectable from direct sequence alignment between *Caenorhabditis* and *Steinernema* but can be found when analyzing genus-level conservation and using conserved gene expression and gene-motif association between orthologs. Further analysis will be required to assess whether these motifs form a phylum-wide core kernel of regulatory relationships or are restricted to the last common nematode ancestor of these two genera.

## Materials and methods

### Strain culturing and maintenance

*S. carpocapsae* (strain All), *S. scapterisci* (strain FL), *S. feltiae* (strain SN), *S. glaseri* (strain NC), and *S. monticolum* (Mount Jiri strain) were reared and maintained using standard methods [70] (Fig. 1, Additional file 1: Figure S1). Briefly, five last-instar *Galleria mellonella* waxmoth larvae or a single adult cricket for *S. scapterisci* (American Cricket Ranch, Lakeside, CA, USA) were placed in a 5 cm Petri dish with a 55 mm Whatman 1 filter paper acting as a pseudo-soil substrate in the bottom of the dish. Up to 250 μl containing 500–1,000 IJs suspended in water was evenly distributed on the filter paper. After 7–10 days the insect cadavers were placed on White traps [71]. Waxmoth cadavers infected with *S. glaseri* were placed in a Petri dish partially filled with plaster of Paris and harvested from this, because *S. glaseri* emerge as pre-IJs that will not properly develop if they emerge directly into water [70]. Emerging IJs from all species were harvested, washed for 10 minutes in 0.4 % Hyamine 1622 solution (Fluka, Switzerland), and rinsed three times with water.

### Isolation of DNA and RNA

To harvest bulk genomic DNA and IJ RNA, IJs from each species were washed in 0.4 % Hyamine, rinsed three times, and acclimated in Ringer’s solution for 15–30 minutes prior to nucleic acid collection. For DNA extraction, a Wizard Genomic DNA Purification Kit (Promega, Madison, WI) was used following the manufacturer’s protocol. The genomic DNA was then treated with RNase A to remove any RNAs present in the sample. For RNA extractions, the nematodes were snap-frozen in liquid nitrogen in ~100 μL aliquots and stored at −80 °C. Worms were then freeze-thawed three or four times to break the cuticle before extracting bulk RNA. Bulk RNA was then extracted using a phenol-chloroform extraction using Trizol (Invitrogen, Carlsbad, CA). This sample was treated with DNase to remove lingering DNA and then poly-A selected to isolate eukaryotic messenger RNA, reducing if not removing bacterial contamination. To isolate embryonic, L1, and adult stage-specific RNA from *S. carpocapsae*, 1,000–2,000 IJs were placed onto 10 cm lipid agar plates seeded with overnight cultures of *Xenorhabdus nematophila* (strain ATCC 19061). These cultures were allowed to grow for ~42 hours to collect young adults, or ~68 hours to collect embryos from mature gravid females. Gravid females were collected from the plates by adding enough distilled water to the cover the surface of the plates, swirling the plates by hand to lift the nematodes into suspension, and pouring them into conical tubes. These were then pelleted by gentle centrifugation and rinsed several times with distilled water until the supernatant was clear. The nematodes were placed in separate 15 mL conical tubes in 7 mL aliquots, and topped off to 15 mL with bleach solution (16.6 mL of 12 % bleach, 5 mL of 1 M KOH, and 80 mL of distilled water). Eggs were harvested by bleaching the nematodes until all nematode tissue was dissolved, leaving only the eggs. These embryos were then either harvested for total RNA as described above, or they were allowed to hatch to L1s in Ringer’s solution over a period of ~30 hours before harvesting the total RNA. To isolate embryonic, L1, and adult stage-specific RNA from *S. feltiae*, 1,000–2,000 IJs were placed onto 10 cm lipid agar plates seeded with overnight cultures of *Xenorhabdus bovienii* (Akhurst and Boemare ATCC 35271). These cultures were allowed to grow for ~36 hours to collect adults or ~55 hours to collect embryos from gravid females. To collect L1s, we waited until all embryos hatched, which was ~24 hours. The same bleaching procedure was followed to harvest embryos and L1s as for *S. carpocapsae*. To isolate embryonic, L1, and adult stage-specific RNA from *C. elegans* (N2 strain) worms were placed onto 10 cm nematode growth media (NGM) plates seeded with overnight cultures of *Escherichia coli* (OP50 strain). To these, 200 uL aliquots of OP50 were added every day. Plates with lots of gravid adults were bleached according to the guide for maintenance of *C. elegans* in Wormbook [72]. The embryos were either collected to harvest embryos, placed in Ringer’s solution for ~20 hours to harvest L1s, or plated on fresh NGM plates seeded with *E. coli* OP50 and collected ~47 hours later to harvest young adults.

### Genomic and RNA-seq library construction

The genomic library was constructed using an Illumina Paired-End DNA Sample Preparation Kit according to the manufacturer’s instructions. Briefly, 3 μg of genomic DNA were fragmented using nebulization. The fragments were end-repaired, 3′-adenylated, and ligated to Illumina’s paired-end adaptors. The ligation products were size-selected on an agarose gel to yield fragments of approximate length of 350 bp. These fragments were then PCR-amplified to produce the finished library. The mate-pair or “jumping” library was prepared using an Illumina Mate Pair Library Preparation Kit v2. Briefly, 7.5 μg of genomic DNA was fragmented using a HydroShear device (Genomic Instrumentation Services, Foster City, CA) to generate fragments of ~2.2 kb. Following end repair and biotinylation, the 2.2 kb fragment was gel-purified and circularized. Circular DNA was fragmented using a Bioruptor NGS (Diagenode, Denville, NJ) and biotin-containing fragments were isolated using Dynabeads (Invitrogen). The fragments were end-repaired, 3′-adenylated, and ligated to NEBNext Multiplex Adaptors (NEB, Ipswich, MA). The ligation products were PCR-amplified and size-selected using AMPure XP beads (Beckman Coulter, Brea, CA) to generate the finished library of approximately 450 bp in length. Genomic libraries were sequenced on an Illumina Genome Analyzer IIx sequencer in paired-end mode with the read length of 76 bp. The jumping library was sequenced on an Illumina HiSeq2000 in paired-end mode with the read length of 100 bp (Additional file 2: Table S20).

The first set of RNA samples, which was used for genome annotation of all genomes, was prepared from 10 μg of total RNA from IJs, poly(A)-selected, and libraries constructed using a standard unstranded protocol [68, 73]. Libraries were quantified using a Qubit fluorometer (Invitrogen) and size distributions were verified using an Agilent Bioanalyzer and the High Sensitivity DNA Kit. These RNA-seq libraries were sequenced on the Illumina Genome Analyzer IIx sequencer in paired-end mode to a read length of 76 bp (Additional file 2: Table S21). The second set of RNA-seq samples, which was used for stage-specific gene expression analyses in *S. carpocapsae* and *S. feltiae*, was prepared from 5–30 μg of total RNA, poly(A)-selected using a Dynabeads mRNA DIRECT Kit (Invitrogen), and fragmented with a hydrolysis buffer containing magnesium ions [73]. Double-stranded cDNA was prepared from the mRNA fragments using Invitrogen’s SuperScript Double-Stranded cDNA Synthesis Kit. During the second strand of reverse transcription, dUNTP (Applied Biosystems, Foster City, CA) was added to label the second strand (stranded protocol), and the libraries were prepared following the Myer’s Lab ChIP-seq protocol version 2011 with Illumina sequencing adapters. The libraries were sequenced on either the Illumina HiSeq 2000 or the NextSeq 500 sequencer in single-end mode to a read length of 50 bp or 75 bp, respectively (Additional file 2: Table S14). Reads for RNA-seq samples used for the gene expression analysis and gene expression tables were submitted to Gene Expression Omnibus (GEO) under the accession number [GSE68588].

### Genome assembly

The genomic libraries were built, sequenced, assembled, filtered, and repeat-masked as previously described [68] using Velvet 1.2.07 and RepeatModeler 1.0.5, RepeatMasker 3.0.3, recon 1.70, and RepeatScout 1.0.5 (Table 1). The genomes and gene annotations are available at [74].

The Whole Genome Shotgun project for *S. carpocapsae* has been deposited at DDBJ/EMBL/GenBank under the accession [AZBU00000000]. The version described in this paper is version AZBU01000000.

The Whole Genome Shotgun project for *S. feltiae* has been deposited at DDBJ/EMBL/GenBank under the accession [AZBV00000000]. The version described in this paper is version AZBV01000000.

The Whole Genome Shotgun project for *S. glaseri* has been deposited at DDBJ/EMBL/GenBank under the accession [AZBX00000000]. The version described in this paper is version AZBX01000000.

The Whole Genome Shotgun project for *S. monticolum* has been deposited at DDBJ/EMBL/GenBank under the accession [AZHV00000000]. The version described in this paper is version AZHV01000000.

The Whole Genome Shotgun project for *S. scapterisci* has been deposited at DDBJ/EMBL/GenBank under the accession [AZBW00000000]. The version described in this paper is version AZBW01000000.

### Transcriptome assembly and genome annotation

IJ-stage, paired-end 75 bp, unstranded RNA-seq data sequenced to an average depth of 76 million reads for *S. feltiae*, *S. glaseri*, *S. monticolum*, and *S. scapterisci*, and embryo, L1, IJ, and adult stage data for *S. carpocapsae* were de novo assembled into expressed sequence tags (ESTs) with Oases 0.2.6 as previously described [75], with the following options: -m 23, -M 59, -s 4, -ins_length. To annotate each genome, ESTs were mapped onto the genome with BLAT 3.4 and these used as hints for gene finding using Augustus 2.6 with *C. elegans* settings (options: --species = caenorhabditis, --gff3 = on, --alternatives-from-evidence = true, --uniqueGeneId = false, --protein = on, --codingseq = on, --noInFrameStop = true, --UTR = on, --hintsfile) [76]. Separately, RNA-seq reads were mapped onto the genome using TopHat 1.4 [77] to find novel transcripts using Cufflinks 2.0.2 [78] (Table 1, Additional file 2: Table S22, Additional file 7), which is described in more detail in a later section below.

### Filtering bacterial symbiont DNA and other bacterial DNA contaminants from genomes

Protein sequences coded by intronless Augustus-predicted genes (putative bacterial contamination) were compared to a database using blastp in Blast2GO [79] to determine the identities of the bacterial contaminants present in the respective nematode genomes (Additional file 1: Figure S2). Assembled bacterial genomes matching the species blast results were obtained from GenBank, and their sequences were compared to the respective nematode genomes with BLAT 3.4, and removed from the nematode assemblies when the sequence match was >94 % identical [80]. After filtering out bacterial DNA contamination, the genome annotations were repeated for each assembly using Augustus.

### Orthology analyses

To study the evolution of gene families across nematodes, we used the available predicted protein datasets from WormBase release WS225 — *Brugia malayi*, *Caenorhabditis elegans*, *Meloidogyne hapla*, *Pristionchus pacificus*, and *Trichinella spiralis* [21–23, 26, 27]. We also included the *Ascaris suum* and *Bursaphelenchus xylophilus* predicted proteome datasets from WormBase release WS229 [24, 25]. We also used the *Panagrellus redivivus* genome assembly prior to its WormBase release [28]. For out-group and comparative analysis we used the predicted protein dataset of the *Nasonia vitripennis* (v1.2) genome project, obtained from the NCBI/NIH repository [29] (Fig. 2a–c, Additional file 1: Figures S3–S10). Version 1.4 of the OrthoMCL pipeline was used to cluster proteins into families of orthologous genes, with default settings and the BLAST parameters recommended in the OrthoMCL documentation [81] (Fig. 2, Additional file 2: Table S1).

### Protein domain analyses

To evaluate the prevalence of protein domains in the proteome of *Steinernema carpocapsae* and other species, we used the hmmscan program from the latest version of HMMER (3.0) software package, which implements probabilistic profile hidden Markov models [82]. We set our threshold *E*-value criterion at 10^−6^, so that no known false-positive matches would be detected in assigning Pfam domain identities. We ran this analysis on the proteomes mentioned above and filtered out splice isoforms from the *C. elegans* proteome.

### Gene tree analyses

Some protein families were further explored by evaluating gene trees either with whole protein sequences or by protein domain sequences. To do these analyses we aligned protein sequences using MUSCLE [83]. Aligned protein sequences were then evaluated by distance analysis using the JTT matrix and a subsequent Neighbor-joining tree was created using the PHYLIP software package version 3.68, using the protdist and neighbor programs, and seqboot where bootstrap values were reported [84] (Fig. 2d, Additional file 1: Figure S11).

### Supermatrix construction and whole genome phylogenetic analysis

The orthology analysis above resulted in 3,885 strictly orthologous genes (1:1 conservation across all steinernematid species and the out-group, *P. redivivus*). These strict orthologs were then compiled and used for the supermatrix construction and subsequent phylogenetic analysis. Because alignment accuracy greatly influences phylogenetic analyses and an earlier study on *Steinernema* phylogeny shows that there can be greater topological variation due to different alignment construction parameters than owing to the methods used to generate the phylogenies [85, 86], we took a very conservative approach to generating the amino acid sequence alignments. Accordingly, each gene was first aligned separately in MAFFT v6.821b [87]. The L-INS-i algorithm was chosen because it is the most accurate setting in MAFFT for datasets containing fewer than 200 species [87]. Because this analysis incorporated more genes (3,885 per species) than can reasonably be checked by eye, we used GBlocks v0.91 [88] to objectively eliminate highly divergent and ambiguously aligned regions of the transformation series [89–91]. Using the batch feature of GBlocks we applied strict settings: four of the six species’ amino acids were required to make a conserved position for a column, five of the six species’ amino acids were required to create a flank position, ten conserved amino acids were required to make a block, eight consecutive non-conserved amino acids was the maximum allowed, and all gaps were removed.

GBlocks identified sequence divergence and alignment ambiguity problems that led us to remove 14 genes from the analysis. Prior to the GBlocks analysis a supermatrix of all of the genes contained 2,924,577 amino acids; the optimized alignment was reduced to 1,320,306 amino acids, a 45 % reduction. GBlocks output was used to concatenate the individual gene files into a supermatrix.

We constructed phylogenetic trees in PAUP* v4.0b10 [90] under the parsimony optimality criterion. The tree search parameters for the supermatrix were an exhaustive parsimony search enforcing a monophyletic root. The result was a separate tree file for each gene and another for the supermatrix. We inferred nodal support by bootstrap analysis [91] of the supermatrix in PAUP* with 500 repetitions using a heuristic search with randomized additions. The parsimony analysis of the supermatrix resulted in only one best tree (Fig. 1b). The bootstrap values were all 100 on each node, suggesting that the data provide strong support for the solution. The tree that was supported by the largest number of genes was the same tree that was the most parsimonious solution for the supermatrix (data not shown).

### Analysis of genome completeness

Genome completeness was determined by clustering *S. carpocapsae*, *S. feltiae*, *S. glaseri*, *S. monticolum*, and *S. scapterisci* protein sets with a core set of highly conserved eukaryotic proteins (Core Eukaryotic Gene Mapping Approach, CEGMA) using OrthoMCL 1.4 as previously described [28, 73, 81, 92]. The percentages of genome completeness for each species was found by dividing the number of proteins that were orthologous to CEGMA proteins by the total number of CEGMA proteins (Additional file 2: Table S2).

### Gene expression analyses

Stranded, single-ended 50 bp RNA-seq reads from the embryonic, L1, IJ, and adult stages of *S. feltiae*, *S. carpocapsae*, and *C. elegans* sequenced to an average depth of 22, 30, and 33 million reads respectively were trimmed to 35 bp to remove low quality bp (Additional file 2: Table S14). Prior to read mapping, transcriptome indexes were prepared for *S. carpocapsae*, *S. feltiae*, and *C. elegans* (WS220) using the RSEM command (version 1.2.12) rsem-prepare-reference [93]. Reads were mapped to each respective species’ annotations using bowtie 0.12.8 with the following options: -S, --offrate 1, -v 1, -k 10, --best, --strata, -m 10 [94]. Gene expression was quantified using the RSEM command, rsem-calculate-expression, with the following options: --bam, --fragment-length-mean [93]. We used EdgeR to analyze genes that were DE during the developmental time course of each species, and we considered a gene to be DE if it had an FDR < 1 × 10^−5^ and a fold change > 4× [95]. DE genes were K-means clustered into eight clusters (Fig. 3a, Additional file 4) using Cluster 3.0 [96], and visualized with JavaTree View [97]. The optimal K for clustering was found using the Akaike information criterion. DE gene clusters were functionally annotated using Blast2GO’s Fisher’s exact test [80].

### Finding novel genes and isoforms using Cufflinks and Cuffmerge

Unstranded paired-end RNA-seq data collected from four *S. carpocapsae* developmental stages (embryo, L1, IJ, adult) were aligned to the *S. carpocapsae* genome using TopHat 1.4.0 and Bowtie 0.12.8 with the following options: -r 50, –G < annotations > [79]. Gene expression for the aligned reads was quantified with Cufflinks 2.0.2 using the following options: -u, -g < annotations>. Transcript annotations from each developmental stage were merged together with Cuffmerge (options: -g < annotations>, -s < genome>) (Additional file 7). The Cuffmerge output showed genes and transcripts that were discovered by Cufflinks but missed by Augustus. The Cufflinks annotations were used in combination with the Augustus annotations to delineate coding versus non-coding sequences in downstream analyses.

Unstranded, paired-end RNA-seq data for the IJ stage in the other species were aligned to their respective genomes using TopHat 1.4.0 and Bowtie 0.12.8 with the following options: -r 50, –G < annotations>. Cuffmerge was not used. Gene expression was quantified with Cufflinks 2.0.2 using the following options: -u, -g < annotations > .

### Multiple genome alignment

Five whole repeat-masked *Steinernema* genomes were aligned using MULTIZ/TBA (multiz-tba.012109). Contigs from the best-assembled genome, *S. carpocapsae*, were concatenated together with 100 bp “N” spacers and used as a reference sequence for the alignment process. The aligned sequences were analyzed with Phast 1.2.1 (phyloFit options: --tree, phastCons options: --target-coverage 0.4, --expected-length 10, --estimate-trees, --nopostprob) to determine regions of sequence conservation across the genomes using setting for *C. elegans* as previously described [68, 98–100]. PhastCons parameters were also varied around those used for *C. elegans* [68], but the *C. elegans* parameters provided a good balance between small and large blocks of conservation. Conserved sequences that matched Augustus and Cufflinks coding sequences or 5′ or 3′ untranslated regions were considered conserved coding sequences, whereas sequences that mapped anywhere else were considered conserved non-coding sequences. DNA from the anterior portion of the Hox cluster (*ceh-13* and *lin-39*) in *S. carpocapsae*, *S. scapterisci*, and *S. feltiae* were also aligned using MUSSA [101] to find conserved regions of their DNA. MUSSA was run with a conservation window size of 30 nucleotides and a nucleotide conservation threshold of 23 nucleotides.

### Gene expression conservation

To determine the degree of gene expression conservation during development between nematode species, we compared gene expression data for four developmental stages in *S. carpocapsae*, *S. feltiae*, and *C. elegans*. Two methods were used for determining conserved gene expression. The first method binarized the expression data using a flexible threshold to sort the genes into stage-specific sets (Additional file 1: Figure S14). We examined the gene expression levels of the 1:1:1 orthologs at four developmental stages and asked if an ortholog that was expressed above an averagely expressed gene (10 FPKM) in a particular set of developmental stages in a nematode species was expressed at least above 5 FPKM in the other nematode species in the same set of developmental stages. If the ortholog was expressed in the same set of developmental stages, it was considered conserved in stage-specific gene expression. If not, stage-specific gene expression was considered to have changed. We used this method to determine the fraction of orthologs that are “on” and “off” in the same developmental stages between species. However, to address whether their expression profiles parallel each other during development, we treated the ortholog expression calculated in transcripts per million (TPM, which is interconvertible with FPKM) during development as vectors, and calculated the cosine similarity (Fig. 3b). The cosine similarity provides a measure of similarity between a pair of vectors: a similarity measure of 1 means that the two vectors are perfectly correlated, whereas a similarity measure of 0 means the vectors are orthogonal (i.e., uncorrelated). We calculated the cosine similarities for each ortholog used in the binary method with developmental stage replicates for each species. We found that orthologs with cosine similarities > 0.95 had extremely similar expression profiles during development, so we set this to be our conservation threshold. This gave us a total of 1,441 orthologs with conserved expression profiles between *S. carpocapsae* and *S. feltiae* (Additional file 6). We sorted these orthologs into stage-specific gene sets by requiring developmental stages to contribute to at least 10 % of the total gene expression during the time course to be considered “on.” Stage-specific gene sets containing more than 30 genes were used for motif finding (e, f, ef, fi, fa, efi, efa, fia, efia).

### Motif discovery

Nine sets of *Steinernema* stage-specific orthologs were chosen for motif mining (Fig. 3c). Conserved non-coding regions ±3 kb or within introns of the genes were obtained by intersecting bed coordinates for the regions upstream of these genes with the genome-wide set of conserved non-coding regions using bedtools/2.15.0 bedintersect [102]. The conserved non-coding bed regions were converted to fasta sequence using bedtools getfasta, filtered for sequences >8 bp, and run through MEME 4.8.1 (settings: -minw 6 -maxw 12 -dna -nmotifs 20-50 -mod zoops -revcomp) to find recurring regulatory motif sequences [103]. We discovered 440 motifs in total across the nine gene sets and searched for them across both the *S. carpocapsae* and *C. elegans* conserved non-coding regions using FIMO with the following settings: --thresh 0.3 --qv-thresh --max-stored-scores 20000000 –bgfile --parse-genomic-coord [104]. We used the WS220 gene annotations and the corresponding conserved regions for *C. elegans* from the UCSC Genome Browser (ce10/WS220: phastConsElements7way.txt) for these analyses. The conserved non-coding regions were produced for *C. elegans* by retaining conserved regions that did not intersect annotated coding regions (bedtools bedintersect, settings = -wa).

### Filtering out redundant and insignificant motifs

Motifs that could not map to any conserved non-coding regions within the q-value threshold (q-value < 0.3) in either species were removed from the analysis. The remaining motif set was compared to itself with TOMTOM to identify redundant motifs, using the following settings: -min-overlap 5 -dist pearson -thresh 0.05 [105]. The redundant motif with the highest MEME e-value of the pair of matching motifs was removed from the analysis. In the end, we were left with 30 non-redundant motifs (Additional file 2: Table S17, Additional file 8).

### Motif-gene association

The final set of non-redundant motifs was associated with the nearest gene models for each species, forming motif-associated gene sets using bedtools closest with the following setting: -d [102].

### Novel motif comparison to WormBase motif database

The final set of 30 motif position weight matrices was compared to 5,512 motifs from WormBase [106, 107] with TOMTOM using the following settings: -min-overlap 5 -dist pearson -evalue -thresh 1.0. Out of 30 motifs, 24 matched WormBase motifs with a *p*-value < 1e^−4^ and an e-value < 0.5 (Additional file 2: Table S18).

### Motif conservation

GO term enrichments were determined for each *S. carpocapsae* and *C. elegans* motif-associated gene set using the Fisher’s exact test in Blast2GO [80]. Motif-associated GO terms with FDRs < 0.05 and that were shared between *S. carpocapsae* and *C. elegans* were considered for the analysis (Fig. 6b, c; Additional files 9 and 10).

### Conserved GO term network generation

Enriched motif-associated GOs (MAGs) shared between *S. carpocapsae* and *C. elegans* were analyzed for the number and percentage of motif-associated 1:1 orthologs shared between them. MAGs that shared 30 % 1:1 orthologs were involved in biological processes under or related to the parent terms such as neurogenesis, embryogenesis, and muscle development. Thus, we focused on GO terms related to these particular processes and generated networks by placing shared 1:1 ortholog targets from related GO terms and the putative conserved motifs that regulate them into three networks: a neurogenesis-related network, an embryonic-related network, and a muscle-related network. The supplemental figures show all the conserved motif–gene associations regardless of motif and gene node degree, while the main figures show all nodes that had degrees greater than 5 (Fig. 6b, c, Additional file 1: Figure S20, Additional files 11, 12, and 13). The motifs and ortholog associations within these networks are conserved between *S. carpocapsae* and *C. elegans.* Motif locations around the gene models were investigated around interesting orthologs, such as *egl-44* and z*ag-1*, to see if the motif sites are conserved in their location or have changed over time (Fig. 6d, Additional file 1: Figure S21).

### Randomized GO term control network

To verify that the motif-associated GO term enrichments we obtained were not due to chance, we created 100 randomized GO term sets by shuffling all of the annotated *S. carpocapsae* gene GO terms that were derived from Blast2GO. We reassigned the GO term sets to new genes that were previously annotated. Unannotated genes were not assigned a randomized GO term set. We applied Fisher’s exact test to all 30 MAG sets using these randomized GO sets (30 motifs × 100 randomizations = 3,000 Fisher’s exact tests in total), and the GO enrichment results for the neuronal, embryo, and muscle GO terms were analyzed. We did not recover enrichments for any GO terms associated with these terms with FDRs < 0.05.

## Additional files

Additional file 1:
**Supplementary figures and legends.** (PDF 3646 kb) Additional file 2:
**Supplementary tables and legends.** (PDF 1760 kb) Additional file 3:
**1:1** ***C. elegans*** **gene identifiers for the 1:1 orthologs conserved across the phylum Nematoda.** These represent putative phylogenetic markers though their informative value remains to be tested. (TXT 7 kb) Additional file 4:
**Genes differentially expressed during** ***S. carpocapsae*** **development.** Lists 4,557 DE genes (differential gene expression cutoff: FDR < 10^−5^ and fold change > 4×) from the eight DE gene expression clusters, and their gene expression levels (FPKM) during *S. carpocapsae* development (Fig. 3a). The far right column indicates which cluster each gene belongs to. Cluster A genes – genes with high expression in the L1, IJ, and adult stage. Cluster B genes - genes with high expression in the L1 and medium/low expression in the IJ stage. Cluster C genes – genes with high expression in the L1 and IJ stage. Cluster D genes – genes with high expression in the IJ stage. Cluster E genes - genes with high expression in the adult stage and medium/low expression in the L1 stage. Cluster F genes - genes with high expression in the L1 and adult stage. Cluster G genes - genes with high expression in the embryo stage. Cluster H genes – genes with high expression in the L1 stage. (TXT 166 kb) Additional file 5:
***S. carpocapsae*** **isoform similarity.** *S. carpocapsae* transcript isoform pairs (where both isoforms have a summed expression > 1 TPM during developmental time course), their isoform similarities (cosine similarities), and the transcript isoform expression (TPM) values of each of the isoforms during development in replicates. Isoforms that had summed expression < 1 TPM were removed from the analysis. (TXT 423 kb) Additional file 6:
***S. carpocapsae*** **and** ***S. feltiae*** **orthologs with conserved expression (cosine similarity > 0.95).** Lists 1,438 *S. carpocapsae* orthologs that have conserved expression with *S. feltiae* during nematode development. The file includes the gene ID of *S. carpocapsae* (column 1), the ortholog expression similarity (cosine similarity, column 2), the expression values (in TPM) of the *S. carpocapsae* and *S. feltiae* orthologs during the time course in replicates (columns 3–18), and the stage(s) that the ortholog expression is conserved in (column 19). (TXT 171 kb) Additional file 7:
**Additional Cufflinks gene and isoform annotations for** ***S. carpocapsae*** **.** The file was generated by combining Cufflink’s transcript annotations for four developmental stages (embryo, L1, IJ, and adult) with the Augustus-predicted gene annotations (.gtf format). Gene and isoform IDs beginning with “CUFF” were predicted by Cufflinks, whereas ones beginning with “L596_” were predicted by Augustus. The Augustus annotations here match the WormBase gene annotations for *S. carpocapsae*. (GTF 55690 kb) Additional file 8:
**MEME motif position weight matrices derived from the final 30 conserved stage-specific gene sets.** These motifs were derived from conserved non-coding regions ±3 kb of *S. carpocapsae* orthologs that have conserved stage-specific expression profiles during development between *S. carpocapsae* and *S. feltiae.* The motif IDs in the file are numbered from 1 to 30. In parentheses next to each motif number is the developmental stage-specific gene set the motif was derived from (e, f, ef, fi, fa, efi, efa, fia, efia) and its old MEME ID. (TXT 17 kb) Additional file 9:
***S. carpocapsae*** **and** ***C. elegans*** **predicted regulatory motifs GO term enrichments.** Thirty significant, non-redundant motifs and the 619 GO terms they are enriched in for both *S. carpocapsae* and *C. elegans*. The heat map shows the –log_10_(*p*-value) for each motif-associated GO term. (PNG 1053 kb) Additional file 10:
***S. carpocapsae*** **and** ***C. elegans*** **shared GO term table.** Contains all the motif-associated GO (MAG) terms (FDR < 0.05) that are shared between *S. carpocapsae* and *C. elegans*. (TXT 188 kb) Additional file 11:
**Embryonic development network file.** Contains motifs that have a conserved association with embryonic development-related genes (See “Methods” regarding motif conservation) in both *S. carpocapsae* and *C. elegans*. The first column contains the motif ID, the second column contains the edge weight, and the third and fourth columns contain *C. elegans* gene IDs in two different formats. (TXT 6 kb) Additional file 12:
**Neurogenesis/axonogenesis network file.** Contains motifs that have a conserved association with neurogenesis-related genes (See “Methods” regarding motif conservation) in both *S. carpocapsae* and *C. elegans*. The first column contains the motif ID, the second column contains the edge weight, and the third and fourth columns contain *C. elegans* gene IDs in two different formats. (TXT 19 kb) Additional file 13:
**Muscle development network file.** Contains motifs that have a conserved association with muscle development-related genes (See “Methods” regarding motif conservation) in both *S. carpocapsae* and *C. elegans*. The first column contains the motif ID, the second column contains the edge weight, and the third and fourth columns contain *C. elegans* gene IDs in two different formats. (TXT 5 kb)

## References

[1] Blaxter ML, De Ley P, Garey JR, Liu LX, Scheldeman P, Vierstraete A (1998). A molecular evolutionary framework for the phylum Nematoda. Nature.

[2] van Megen H, van Den Elsen S, Holterman M, Karssen G, Mooyman P, Bongers T (2009). A phylogenetic tree of nematodes based on about 1200 full-length small subunit ribosomal DNA sequences. Nematology.

[3] Castelletto ML, Gang SS, Okubo RP, Tselikova AA, Nolan TJ, Platzer EG (2014). Diverse host-seeking behaviors of skin-penetrating nematodes. PLoS Pathog.

[4] Dillman AR, Guillermin ML, Lee JH, Kim B, Sternberg PW, Hallem EA (2012). Olfaction shapes host-parasite interactions in parasitic nematodes. Proc Natl Acad Sci U S A.

[5] Kaya HK, Gaugler R (1993). Entomopathogenic nematodes. Annu Rev Entomol.

[6] Dillman AR, Chaston JM, Adams BJ, Ciche TA, Goodrich-Blair H, Stock SP (2012). An entomopathogenic nematode by any other name. PLoS Pathog.

[7] Gaugler R, Kaya HK (1990). Entomopathogenic nematodes in biological control.

[8] Dillman AR, Sternberg PW (2012). Entomopathogenic nematodes. Curr Biol.

[9] Stock SP, Goodrich-Blair HG (2008). Entomopathogenic nematodes and their bacterial symbionts: The inside out of a mutualistic association. Symbiosis.

[10] Castillo JC, Reynolds SE, Eleftherianos I (2011). Insect immune response to nematode parasites. Trends Parasitol.

[11] Hallem EA, Dillman AR, Hong AV, Zhang Y, Yano JM, DeMarco SF (2011). A sensory code for host seeking in parasitic nematodes. Curr Biol.

[12] Davidson EH (2010). Emerging properties of animal gene regulatory networks. Nature.

[13] Stein LD, Bao ZR, Blasiar D, Blumenthal T, Brent MR, Chen NS (2003). The genome sequence of *Caenorhabditis briggsae*: A platform for comparative genomics. PLoS Biol.

[14] Havird JC, Miyamoto MM (2010). The importance of taxon sampling in genomic studies: an example from the cyclooxygenases of teleost fishes. Mol Phylogenet Evol.

[15] Kubatko LS, Degnan JH (2007). Inconsistency of phylogenetic estimates from concatenated data under coalescence. Syst Biol.

[16] Nadler SA, Bolotin E, Stock SP (2006). Phylogenetic relationships of Steinernema Travassos, (Nematoda: Cephalobina: Steinernematidae) based on nuclear, mitochondrial and morphological data. Syst Parasitol.

[17] Rokas A, Williams BL, King N, Carroll SB (2003). Genome-scale approaches to resolving incongruence in molecular phylogenies. Nature.

[18] Zhao L, Zhang N, Ma PF, Liu Q, Li DZ, Guo ZH (2013). Phylogenomic analyses of nuclear genes reveal the evolutionary relationships within the BEP clade and the evidence of positive selection in Poaceae. Plos One.

[19] Holterman M, van der Wurff A, van den Elsen S, van Megen H, Bongers T, Holovachov O (2006). Phylum-wide analysis of SSU rDNA reveals deep phylogenetic relationships among nematodes and accelerated evolution toward crown clades. Mol Biol Evol.

[20] Adams BJ, Peat SM, Dillman AR. Phylogeny and evolution. In: Nguyen KB, Hunt DJ, editors. Entomopathogenic nematodes: Systematics, phylogeny, and bacterial symbionts, Volume 5. Leiden-Boston: Brill; 2007. p. 693–733. Nematology monographs and perspectives.

[21] C. elegans Sequencing Consortium. Genome sequence of the nematode *C. elegans*: a platform for investigating biology. Science. 1998;282:2012–8. 10.1126/science.282.5396.2012 9851916

[22] Dieterich C, Clifton SW, Schuster LN, Chinwalla A, Delehaunty K, Dinkelacker I (2008). The *Pristionchus pacificus* genome provides a unique perspective on nematode lifestyle and parasitism. Nat Genet.

[23] Ghedin E, Wang S, Spiro D, Caler E, Zhao Q, Crabtree J (2007). Draft genome of the filarial nematode parasite *Brugia malayi*. Science.

[24] Jex AR, Liu S, Li B, Young ND, Ross SH, Li Y (2011). *Ascaris suum* draft genome. Nature.

[25] Kikuchi T, Cotton JA, Dalzell JJ, Hasegawa K, Kanzaki N, McVeigh P (2011). Genomic insights into the origin of parasitism in the emerging plant pathogen *Bursaphelenchus xylophilus*. PLoS Pathog.

[26] Mitreva M, Jasmer DP, Zarlenga DS, Wang Z, Abubucker S, Martin J (2011). The draft genome of the parasitic nematode *Trichinella spiralis*. Nat Genet.

[27] Opperman CH, Bird DM, Williamson VM, Rokhsar DS, Burke M, Cohn J (2008). Sequence and genetic map of *Meloidogyne hapla*: a compact nematode genome for plant parasitism. Proc Natl Acad Sci U S A.

[28] Srinivasan J, Dillman AR, Macchietto MG, Heikkinen L, Lakso M, Fracchia KM (2013). The draft genome and transcriptome of *Panagrellus redivivus* are shaped by the harsh demands of a free-living lifestyle. Genetics.

[29] Werren JH, Richards S, Desjardins CA, Niehuis O, Gadau J, Colbourne JK (2010). Functional and evolutionary insights from the genomes of three parasitoid *Nasonia* species. Science.

[30] Clark AG, Eisen MB, Smith DR, Bergman CM, Oliver B, Markow TA (2007). Evolution of genes and genomes on the *Drosophila* phylogeny. Nature.

[31] Kanost MR, Clarke T, Gilbert LI, Iatrou K, Gill S (2005). Proteases. Comprehensive Molecular Insect Science.

[32] Abuhatab M, Selvan S, Gaugler R (1995). Role of proteases in penetration of insect gut by the entomopathogenic nematode *Steinernema glaseri* (Nematoda, Steinernematidae). J Invertebr Pathol.

[33] Balasubramanian N, Hao YJ, Toubarro D, Nascimento G, Simoes N (2009). Purification, biochemical and molecular analysis of a chymotrypsin protease with prophenoloxidase suppression activity from the entomopathogenic nematode *Steinernema carpocapsae*. Int J Parasitol.

[34] McKerrow JH, Brindley P, Brown M, Gam AA, Staunton C, Neva FA (1990). *Strongyloides stercoralis*: identification of a protease that facilitates penetration of skin by the infective larvae. Exp Parasitol.

[35] Toubarro D, Lucena-Robles M, Nascimento G, Costa G, Montiel R, Coelho AV (2009). An apoptosis-inducing serine protease secreted by the entomopathogenic nematode *Steinernema carpocapsae*. Int J Parasitol.

[36] Burman M (1982). *Neoaplectana carpocapsae*: toxin production by axenic insect parasitic nematodes. Nematologica.

[37] Dunphy GB, Rutherford TA, Webster JM (1985). Growth and virulence of *Steinernema glaseri* influenced by different subspecies of *Xenorhabdus nematophilus*. J Nematol.

[38] Dunphy GB, Webster JM (1985). Influence of *Steinernema feltiae* (Filipjev) Wouts, Mracek, Gerdin and Bedding DD136 strain on the humoral and hemocytic responses of *Galleria mellonella* (L) larvae to selected bacteria. Parasitology.

[39] Han R, Ehlers RU (2000). Pathogenicity, development, and reproduction of *Heterorhabditis bacteriophora* and *Steinernema carpocapsae* under axenic *in vivo* conditions. J Invertebr Pathol.

[40] Simóes N, Caldas C, Rosa JS, Bonifassi E, Laumond C (2000). Pathogenicity caused by high virulent and low virulent strains of *Steinernema carpocapsae* to *Galleria mellonella*. J Invertebr Pathol.

[41] James ER, Green DR (2004). Manipulation of apoptosis in the host-parasite interaction. Trends Parasitol.

[42] Trap C, Boireau P (2000). Proteases in helminthic parasites. Vet Res.

[43] Zang X, Maizels RM (2001). Serine proteinase inhibitors from nematodes and the arms race between host and pathogen. Trends Biochem Sci.

[44] Balasubramanian N, Toubarro D, Simoes N (2010). Biochemical study and *in vitro* insect immune suppression by a trypsin-like secreted protease from the nematode *Steinernema carpocapsae*. Parasite Immunol.

[45] Jing Y, Toubarro D, Hao Y, Simoes N (2010). Cloning, characterisation and heterologous expression of an astacin metalloprotease, Sc-AST, from the entomoparasitic nematode *Steinernema carpocapsae*. Mol Biochem Parasitol.

[46] Milstone AM, Harrison LM, Bungiro RD, Kuzmic P, Cappello M (2000). A broad spectrum Kunitz type serine protease inhibitor secreted by the hookworm *Ancylostoma ceylanicum*. J Biol Chem.

[47] Rawlings ND, Barrett AJ, Bateman A (2012). MEROPS: the database of proteolytic enzymes, their substrates and inhibitors. Nucleic Acids Res.

[48] Molehin AJ, Gobert GN, McManus DP (2012). Serine protease inhibitors of parasitic helminths. Parasitology.

[49] Kennedy MW, Corsico B, Cooper A, Smith BO, Kennedy MW, Harnett W (2013). The unusual lipid-binding proteins of nematodes: NPAs, nemFABPs and FARs. Parasitic nematodes: molecular biology, biochemistry, and immunology.

[50] Garofalo A, Klager SL, Rowlinson MC, Nirmalan N, Klion A, Allen JE (2002). The FAR proteins of filarial nematodes: secretion, glycosylation and lipid binding characteristics. Mol Biochem Parasitol.

[51] Hao YJ, Montiel R, Abubucker S, Mitreva M, Simoes N (2010). Transcripts analysis of the entomopathogenic nematode *Steinernema carpocapsae* induced *in vitro* with insect haemolymph. Mol Biochem Parasitol.

[52] Iberkleid I, Vieira P, Engler JD, Firester K, Spiegel Y, Horowitz SB (2013). Fatty acid-and retinol-binding protein, Mj-FAR-1 induces tomato host susceptibility to root-knot nematodes. Plos One.

[53] Campos ML, Kang JH, Howe GA (2014). Jasmonate-triggered plant immunity. J Chem Ecol.

[54] Lawrence T, Willoughby DA, Gilroy DW (2002). Anti-inflammatory lipid mediators and insights into the resolution of inflammation. Nat Rev Immunol.

[55] Stanley D, Miller J. Eicosanoids in invertebrate immunity: an *in vitro* approach. In Vitro Cell Dev Biology Ani. 2006;42:5a–a.

[56] Carton Y, Frey F, Stanley DW, Vass E, Nappi AJ (2002). Dexamethasone inhibition of the cellular immune response of *Drosophila melanogaster* against a parasitoid. J Parasitol.

[57] Park Y, Kim Y (2000). Eicosanoids rescue *Spodoptera exigua* infected with *Xenorhabdus nematophilus*, the symbiotic bacteria to the entomopathogenic nematode *Steinernema carpocapsae*. J Insect Physiol.

[58] Park Y, Kim Y, Putnam SM, Stanley DW (2003). The bacterium *Xenorhabdus nematophilus* depresses nodulation reactions to infection by inhibiting eicosanoid biosynthesis in tobacco hornworms, *Manduca sexta*. Arch Insect Biochem Physiol.

[59] Sulston JE, Schierenberg E, White JG, Thomson JN (1983). The embryonic cell lineage of the nematode *Caenorhabditis elegans*. Dev Biol.

[60] Sulston J, Horvitz HR (1977). Postembryonic cell lineages of the nematode *Caenorhabditis elegans*. Dev Biol.

[61] Sinha A, Sommer RJ, Dieterich C (2012). Divergent gene expression in the conserved dauer stage of the nematodes *Pristionchus pacificus* and *Caenorhabditis elegans*. BMC Genomics.

[62] Wittkopp PJ, Kalay G (2012). Cis-regulatory elements: molecular mechanisms and evolutionary processes underlying divergence. Nat Rev Genet.

[63] Garofalo A, Rowlinson MC, Amambua NA, Hughes JM, Kelly SM, Price NC (2003). The FAR protein family of the nematode *Caenorhabditis elegans* - differential lipid binding properties, structural characteristics, and developmental regulation. J Biol Chem.

[64] Aboobaker A, Blaxter M (2003). Hox gene evolution in nematodes: novelty conserved. Curr Opin Genet Dev.

[65] Aboobaker A, Blaxter M (2010). The nematode story: Hox gene loss and rapid evolution. Adv Exp Med Biol.

[66] Necsulea A, Kaessmann H (2012). Evolutionary dynamics of coding and non-coding transcriptomes. Nat Rev Genet.

[67] Villar D, Flicek P, Odom DT (2014). Evolution of transcription factor binding in metazoans - mechanisms and functional implications. Nat Rev Genet.

[68] Mortazavi A, Schwarz EM, Williams BA, Schaeffer L, Antoshechkin I, Wold B (2010). Scaffolding a *Caenorhabditis* nematode genome with RNA-seq. Genome Res.

[69] Dillman AR, Mortazavi A, Sternberg PW (2012). Incorporating genomics into the toolkit of nematology. J Nematol.

[70] Kaya HK, Stock SP, Lacey L (1997). Techniques in insect nematology. Manual of techniques in insect pathology.

[71] White GF (1927). A method for obtaining infective nematode larvae from cultures. Science.

[72] Stiernagle T. Maintenance of C. elegans. In: The *C. elegans* Research Community, eds. WormBook. 2006. doi/10.1895/wormbook.1.7.1

[73] Mortazavi A, Williams BA, McCue K, Schaeffer L, Wold B (2008). Mapping and quantifying mammalian transcriptomes by RNA-Seq. Nat Methods.

[74] WormBase Parasite. http://parasite.wormbase.org

[75] Schulz MH, Zerbino DR, Vingron M, Birney E (2012). Oases: robust de novo RNA-seq assembly across the dynamic range of expression levels. Bioinformatics.

[76] Stanke M, Diekhans M, Baertsch R, Haussler D (2008). Using native and syntenically mapped cDNA alignments to improve de novo gene finding. Bioinformatics.

[77] Trapnell C, Pachter L, Salzberg SL (2009). TopHat: discovering splice junctions with RNA-seq. Bioinformatics.

[78] Trapnell C, Williams BA, Pertea G, Mortazavi A, Kwan G, van Baren MJ (2010). Transcript assembly and quantification by RNA-Seq reveals unannotated transcripts and isoform switching during cell differentiation. Nat Biotechnol.

[79] Conesa A, Gotz S, Garcia-Gomez JM, Terol J, Talon M, Robles M (2005). Blast2GO: a universal tool for annotation, visualization and analysis in functional genomics research. Bioinformatics.

[80] Kent WJ (2002). BLAT--the BLAST-like alignment tool. Genome Res.

[81] Li L, Stoeckert CJ, Roos DS (2003). OrthoMCL: identification of ortholog groups for eukaryotic genomes. Genome Res.

[82] Finn RD, Clements J, Eddy SR (2011). HMMER web server: interactive sequence similarity searching. Nucleic Acids Res.

[83] Edgar RC (2004). MUSCLE: a multiple sequence alignment method with reduced time and space complexity. BMC Bioinformatics.

[84] Felsenstein J (2005). PHYLIP (Phylogeny Inference Package).

[85] Nguyen KB, Maruniak J, Adams BJ (2001). Diagnostic and phylogenetic utility of the rDNA internal transcribed spacer sequences of Steinernema. J Nematol.

[86] Simmons MP, Muller KF, Webb CT (2011). The deterministic effects of alignment bias in phylogenetic inference. Cladistics.

[87] Katoh K, Misawa K, Kuma K, Miyata T (2002). MAFFT: a novel method for rapid multiple sequence alignment based on fast Fourier transform. Nucleic Acids Res.

[88] Castresana J (2000). Selection of conserved blocks from multiple alignments for their use in phylogenetic analysis. Mol Biol Evol.

[89] Talavera G, Castresana J (2007). Improvement of phylogenies after removing divergent and ambiguously aligned blocks from protein sequence alignments. Syst Biol.

[90] Swofford DL (2002). Phylogenetic analysis using parsimony (*and other methods).

[91] Felsenstein J (1985). Phylogenies and the comparative method. Am Nat.

[92] Parra G, Bradnam K, Korf I (2007). CEGMA: a pipeline to accurately annotate core genes in eukaryotic genomes. Bioinformatics.

[93] Li B, Dewey CN (2011). RSEM: accurate transcript quantification from RNA-seq data with or without a reference genome. BMC Bioinformatics.

[94] Langmead B, Trapnell C, Pop M, Salzberg SL (2009). Ultrafast and memory-efficient alignment of short DNA sequences to the human genome. Genome Biol.

[95] Robinson MD, McCarthy DJ, Smyth GK (2010). edgeR: a Bioconductor package for differential expression analysis of digital gene expression data. Bioinformatics.

[96] de Hoon MJ, Imoto S, Nolan J, Miyano S (2004). Open source clustering software. Bioinformatics.

[97] Saldanha AJ (2004). Java Treeview-extensible visualization of microarray data. Bioinformatics.

[98] Felsenstein J, Churchill GA (1996). A Hidden Markov Model approach to variation among sites in rate of evolution. Mol Biol Evol.

[99] Margulies EH, Blanchette M, Program NCS, Haussler D, Green ED (2003). Identification and characterization of multi-species conserved sequences. Genome Res.

[100] Siepel A, Bejerano G, Pedersen JS, Hinrichs AS, Hou M, Rosenbloom K (2005). Evolutionarily conserved elements in vertebrate, insect, worm, and yeast genomes. Genome Res.

[101] Kuntz SG, Schwarz EM, DeModena JA, De Buysscher T, Trout D, Shizuya H (2008). Multigenome DNA sequence conservation identifies Hox cis-regulatory elements. Genome Res.

[102] Quinlan AR, Hall IM (2010). BEDTools: A flexible suite of utilities for comparing genomic features. Bioinformatics.

[103] Bailey TL, Boden M, Buske FA, Frith M, Grant CE, Clementi L (2009). MEME SUITE: tools for motif discovery and searching. Nucleic Acids Res.

[104] Grant CE, Bailey TL, Noble WS (2011). FIMO: Scanning for occurrences of a given motif. Bioinformatics.

[105] Gupta S, Stamatoyannopoulos JA, Bailey TL, Noble WS (2007). Quantifying similarity between motifs. Genome Biol.

[106] Araya CL, Kawli T, Kundaje A, Jiang LX, Wu BJ, Vafeados D (2014). Regulatory analysis of the *C. elegans* genome with spatiotemporal resolution. Nature.

[107] Gerstein MB, Lu ZJ, Van Nostrand EL, Cheng C, Arshinoff BI, Liu T (2010). Integrative Analysis of the *Caenorhabditis elegans* genome by the modENCODE project. Science.

