# Supplementary material for: Comparative genomics of Steinernema reveals deeply conserved gene regulatory networks
Source: Genome Biol. 2015 Sep 21;16:200. doi: 10.1186/s13059-015-0746-6 (PMC4578762; doi:10.1186/s13059-015-0746-6)
Supplement: Additional file 1: — Supplementary figures and legends. (PDF 3646 kb) [file 13059_2015_746_MOESM1_ESM.pdf]

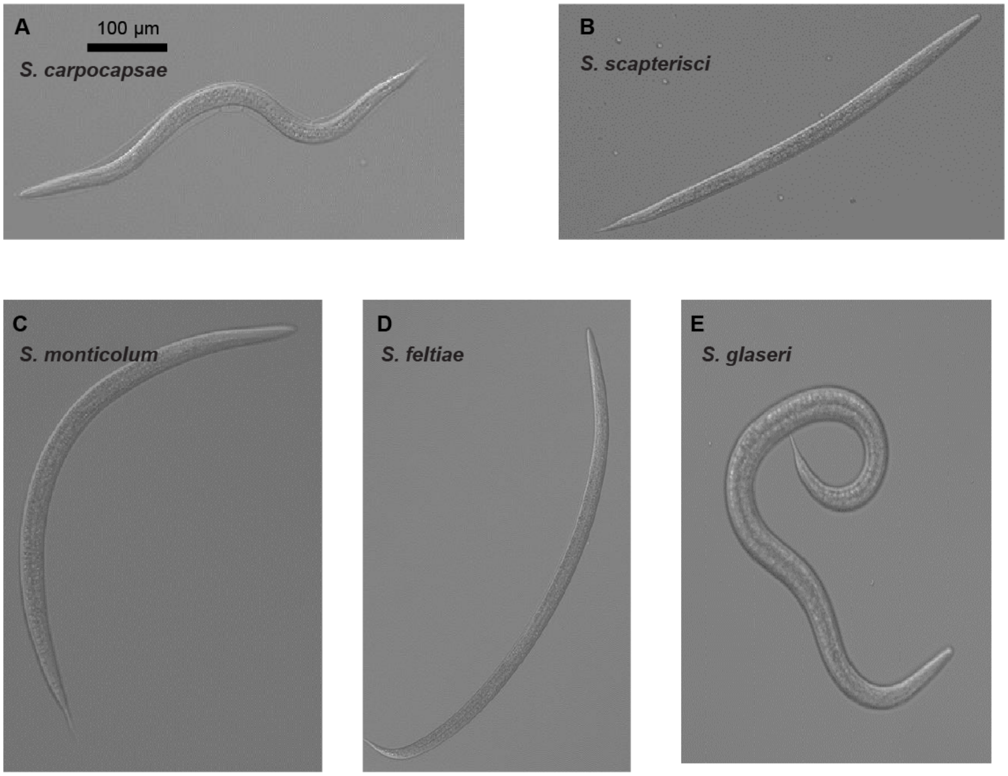

Figure S1. Pictures of the infective juvenile stage of the sequenced *Steinernema* species. **A)** *S. carpocapsae*; **B)** *S. scapterisci*; **C)** *S. monticolum*; **D)** *S. feltiae*; **E)** *S. glaseri*.

A.

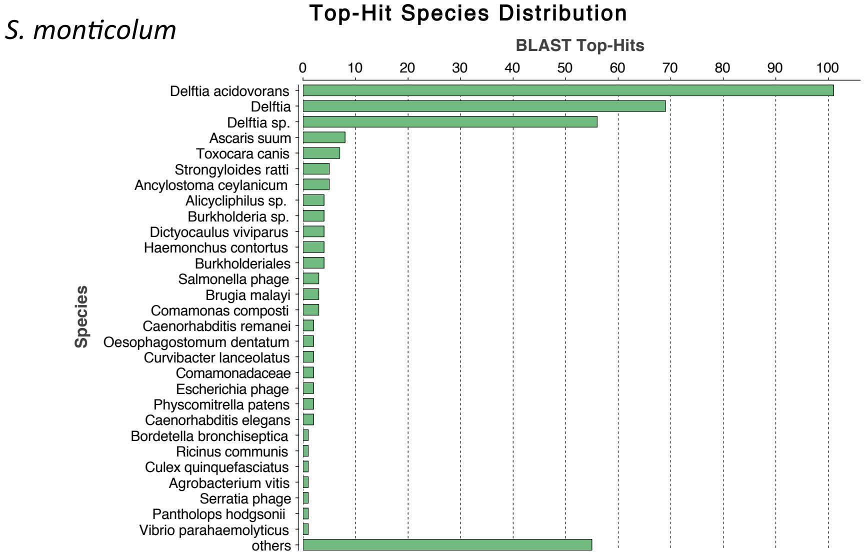

B.

Genome size (Mb)	***carpocapsae***	***scapterisci***	***feltiae***	***glaseri***	***monticolum***
Before bacterial filtration	91.4	112.1	97.4	103.1	131.9
After bacterial filtration	85.6	79.7	82.4	92.9	89.3

Figure S2. Bacterial contamination discovered in *Steinernema* genomes assemblies. A) An example of the bacterial contamination we found across the *Steinernema* genomes. *S. monticolum* single exon gene sequences (putative bacterial genes) were compared to sequences in a BLAST database and annotated with Blast2GO. The plot shows the species with the highest number of blast hits to the *S. monticolum* gene sequences is the bacterial species *Delftia acidovorans*. **B)** Genome assembly sizes of *Steinernema* species before and after filtration of bacterial genomes.

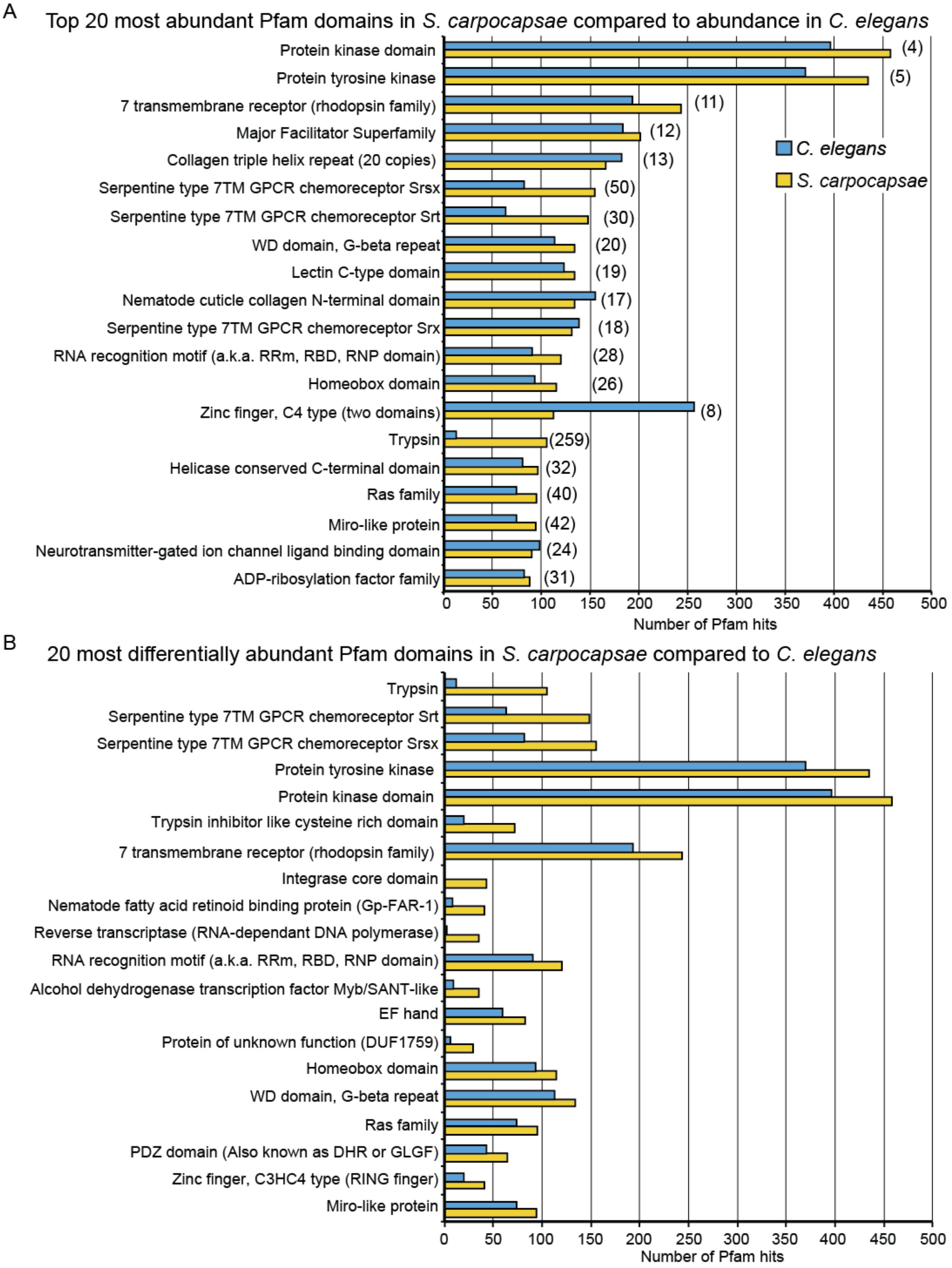

Figure S3. Pfam domain analysis comparing Pfam domains in *C. elegans* and *S. carpocapsae*. Top 20 most abundant Pfam domains present in *C. elegans* and *S. carpocapsae.*

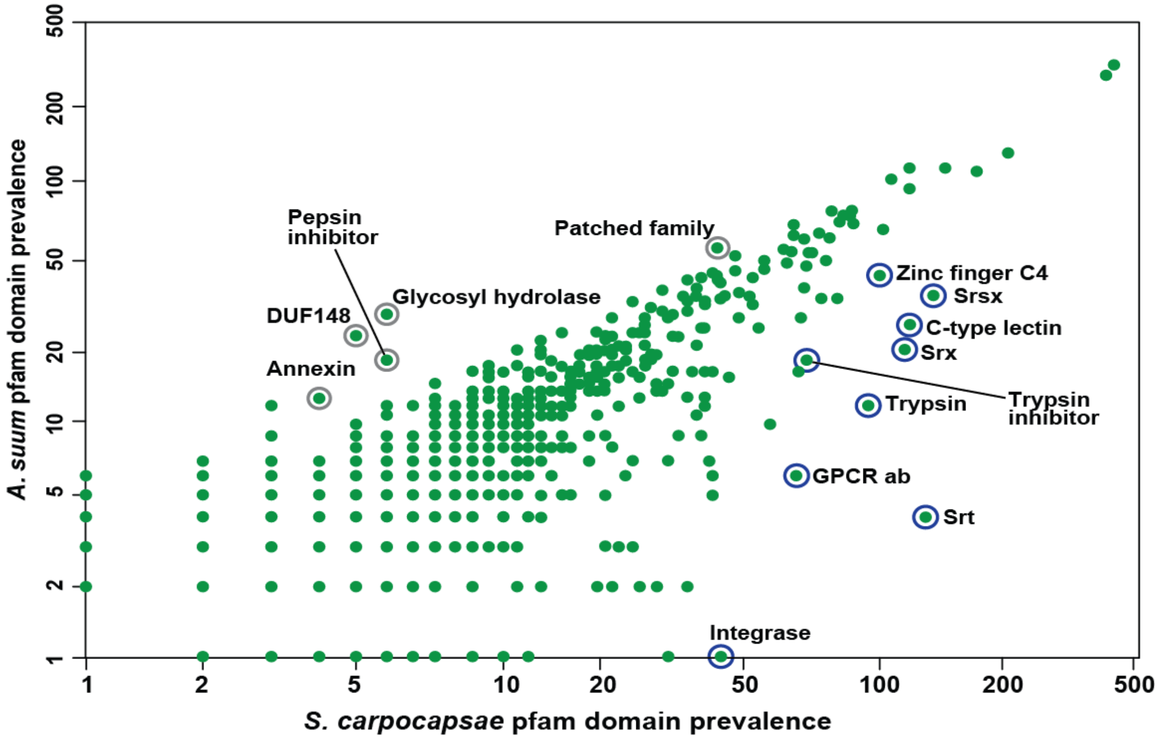

Figure S4. Pfam domain analysis comparing Pfam domains in *A. suum* and *S. carpocapsae*. A scatterplot showing the abundance of Pfam protein family domains in the *A. suum* and *S. carpocapsae* genomes. The most enriched Pfam domains (biggest absolute difference) in *S. carpocapsae* relative to *A. suum* are highlighted in blue while the most enriched Pfam domains in *A. suum* relative to *S. carpocapsae* are highlighted in grey.

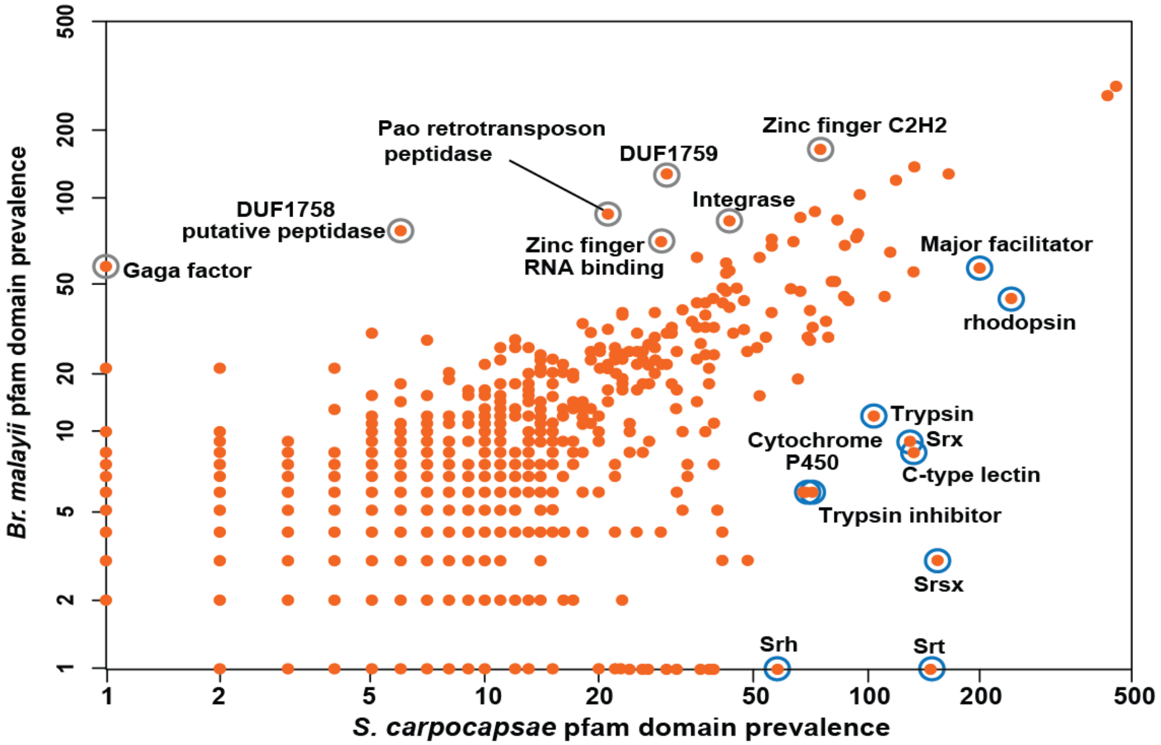

Figure S5. Pfam domain analysis comparing Pfam domains in *B. malayi* and *S. carpocapsae*. A scatterplot showing the abundance of Pfam protein family domains in the *B. malayi* and *S. carpocapsae* genomes. The most enriched Pfam domains (biggest absolute difference) in *S. carpocapsae* relative to *B. malayi* are highlighted in blue while the most enriched Pfam domains in *B. malayi* relative to *S. carpocapsae* are highlighted in grey.

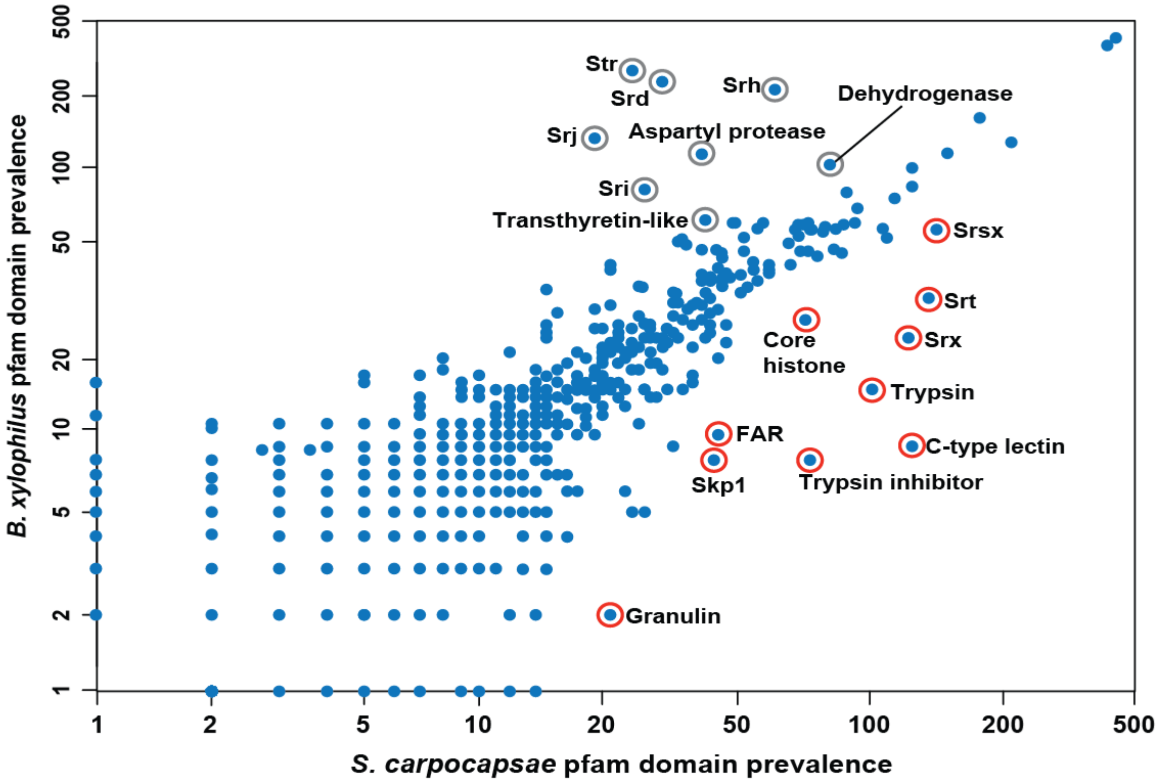

Figure S6. Pfam domain analysis comparing Pfam domains in *B. xylophilus* and *S. carpocapsae*. A scatterplot showing the abundance of Pfam protein family domains in the *B. xylophilus* and *S. carpocapsae* genomes. The most enriched Pfam domains (biggest absolute difference) in *S. carpocapsae* relative to *B. xylophilus* are highlighted in red while the most enriched Pfam domains in *B. xylophilus* relative to *S. carpocapsae* are highlighted in grey.

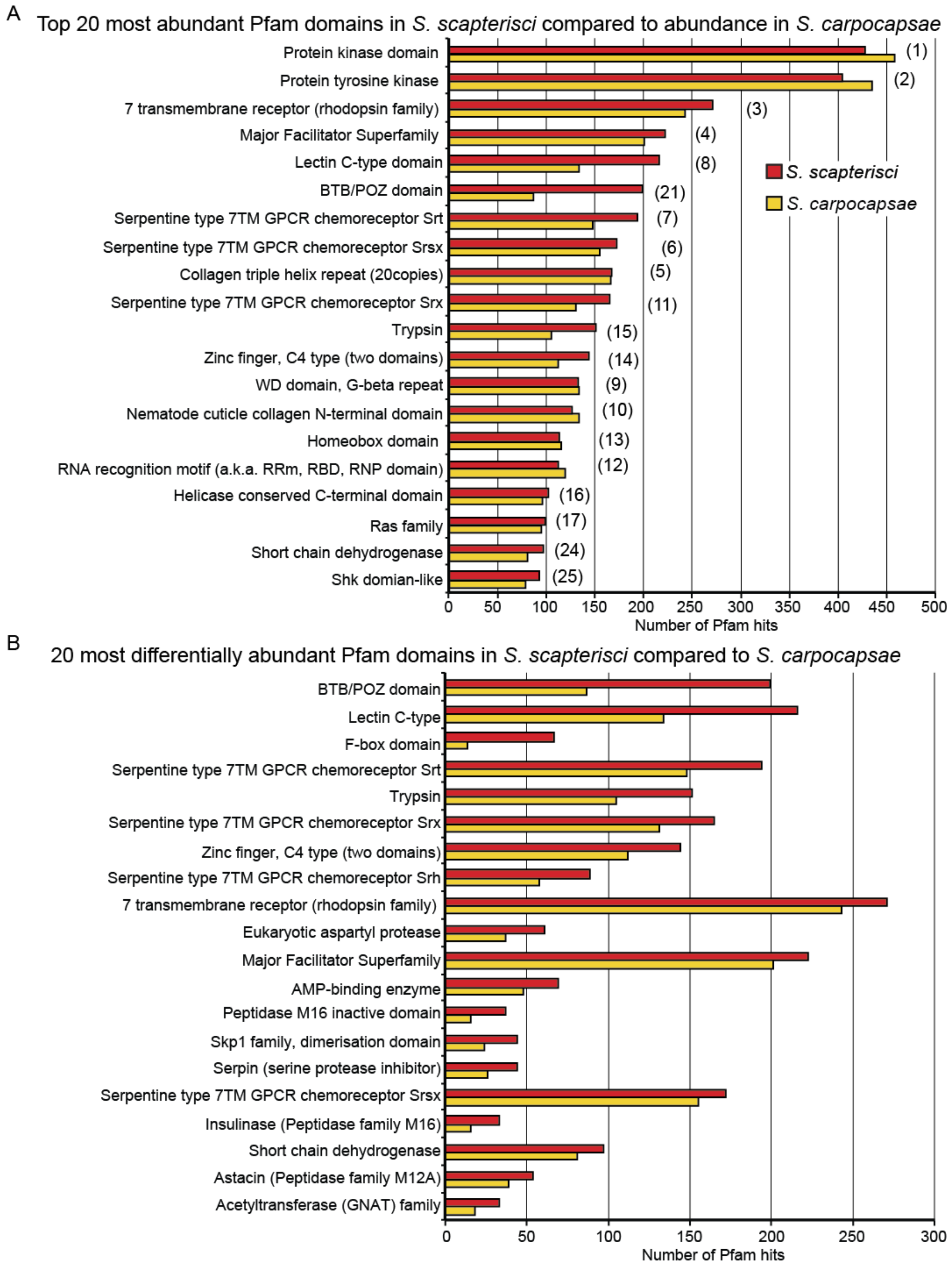

Figure S7. Pfam domain analysis comparing Pfam domains in *S. carpocapsae* and *S. scapterisci*. Top 20 most abundant Pfam domains present in *S. carpocapsae* and *S. scapterisci.*

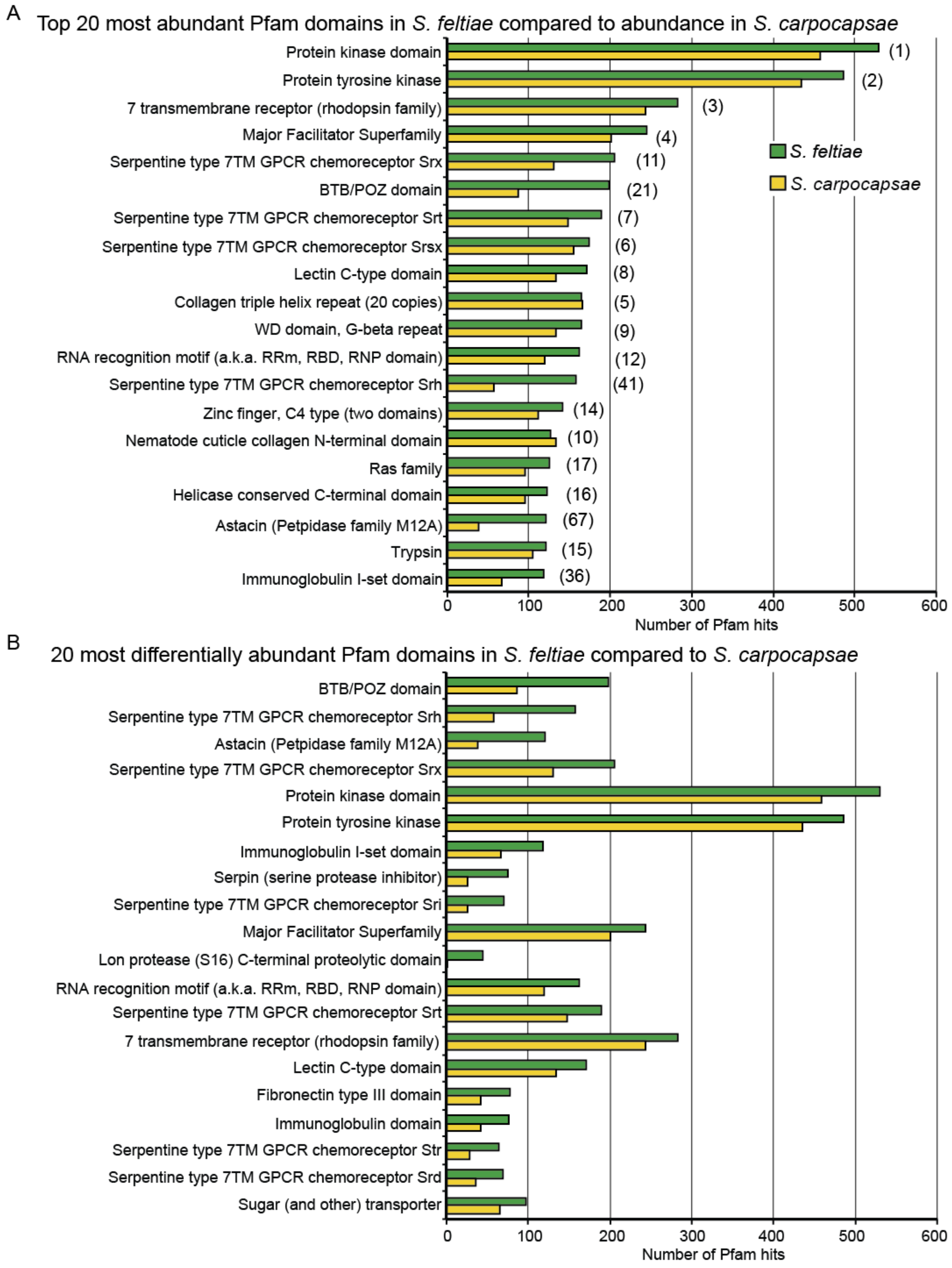

Figure S8. Pfam domain analysis comparing Pfam domains in *S. carpocapsae* and *S. feltiae*. Top 20 most abundant Pfam domains present in *S. carpocapsae* and *S. feltiae.*

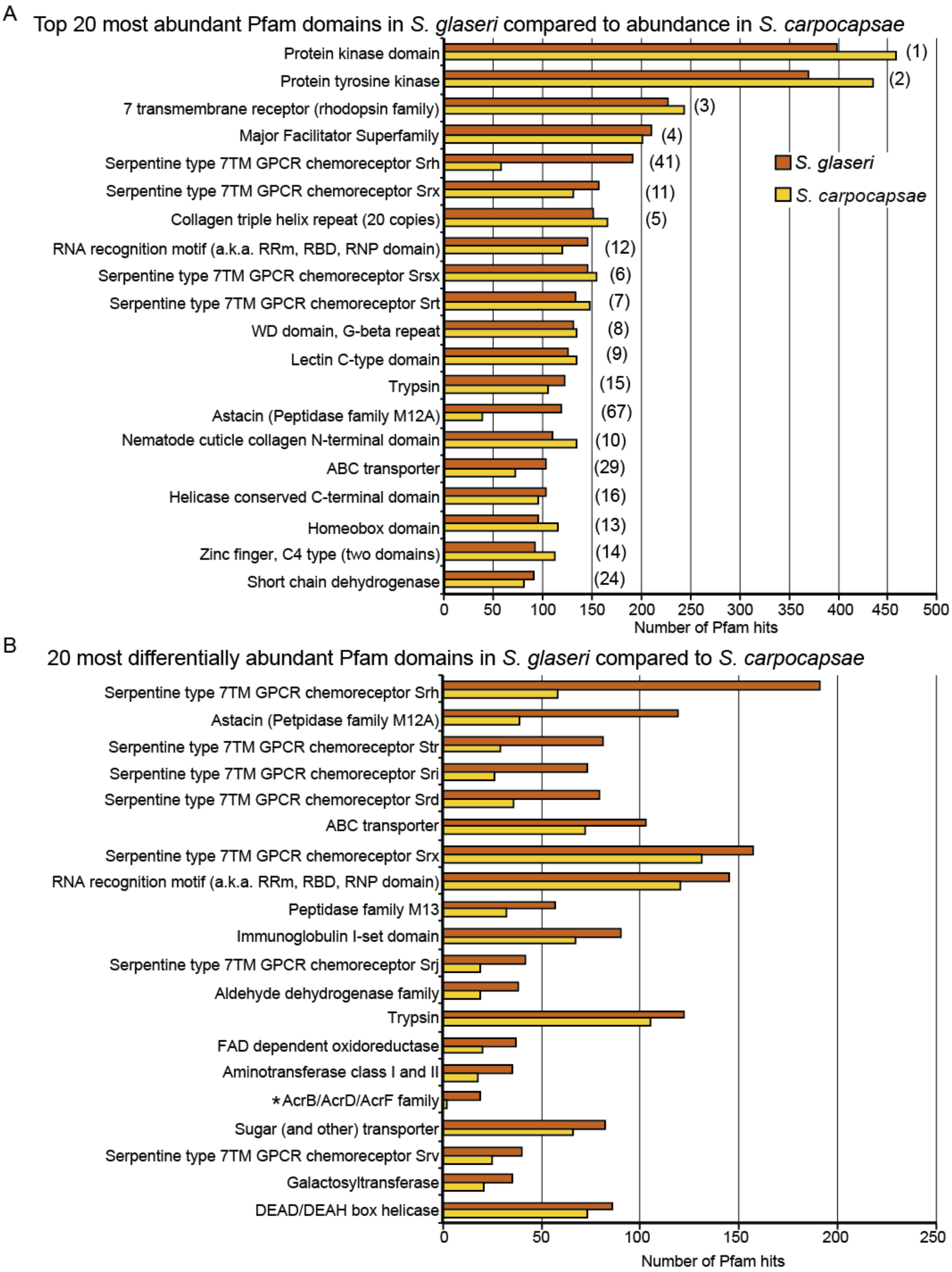

Figure S9. Pfam domain analysis comparing Pfam domains in *S. carpocapsae* and *S. glaseri*. Top 20 most abundant Pfam domains present in *S. carpocapsae* and *S. glaseri.*

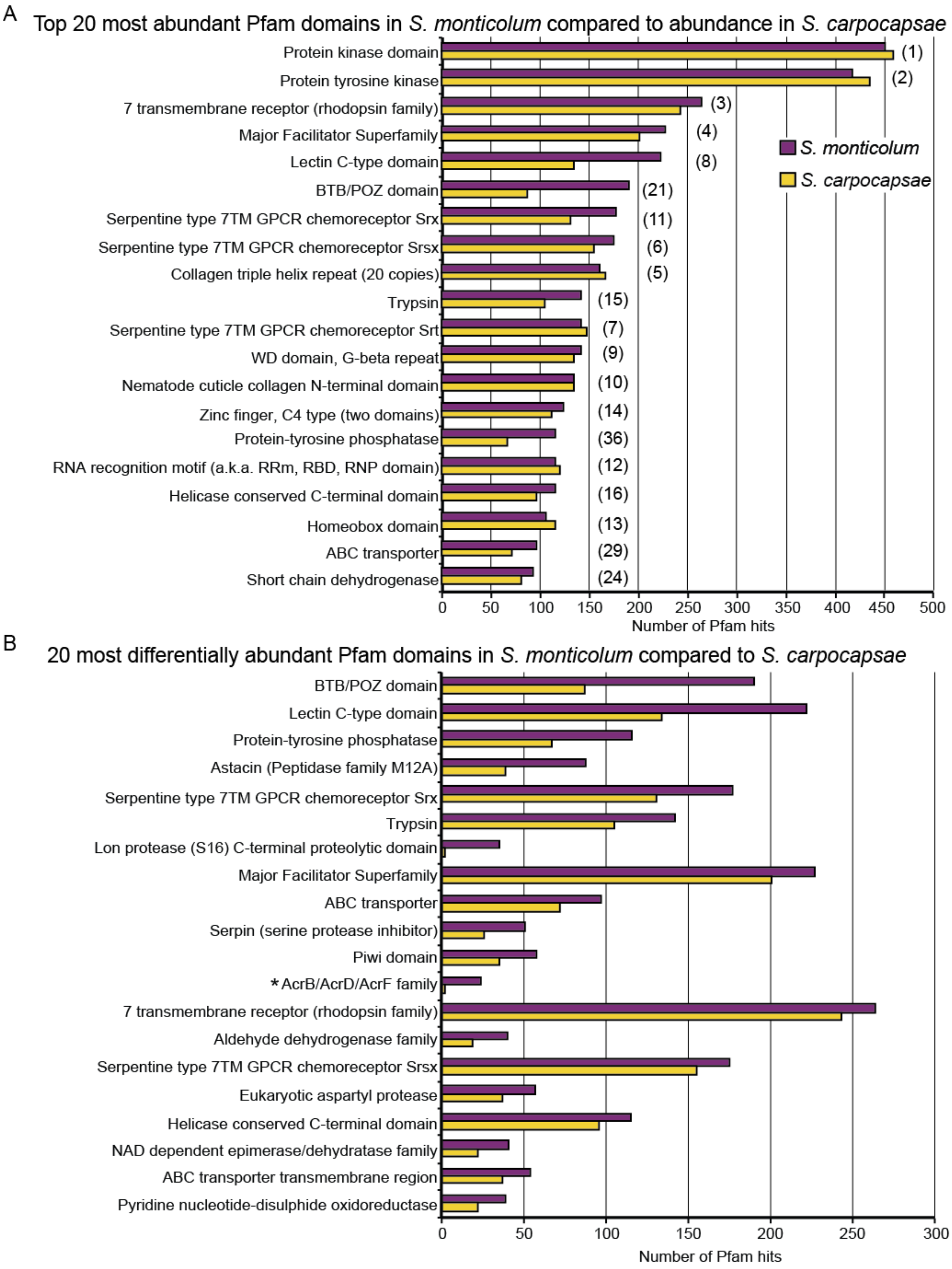

Figure S10. Pfam domain analysis comparing Pfam domains in *S. carpocapsae* and *S. monticolum*. Top 20 most abundant Pfam domains present in *S. carpocapsae* and *S. monticolum.*

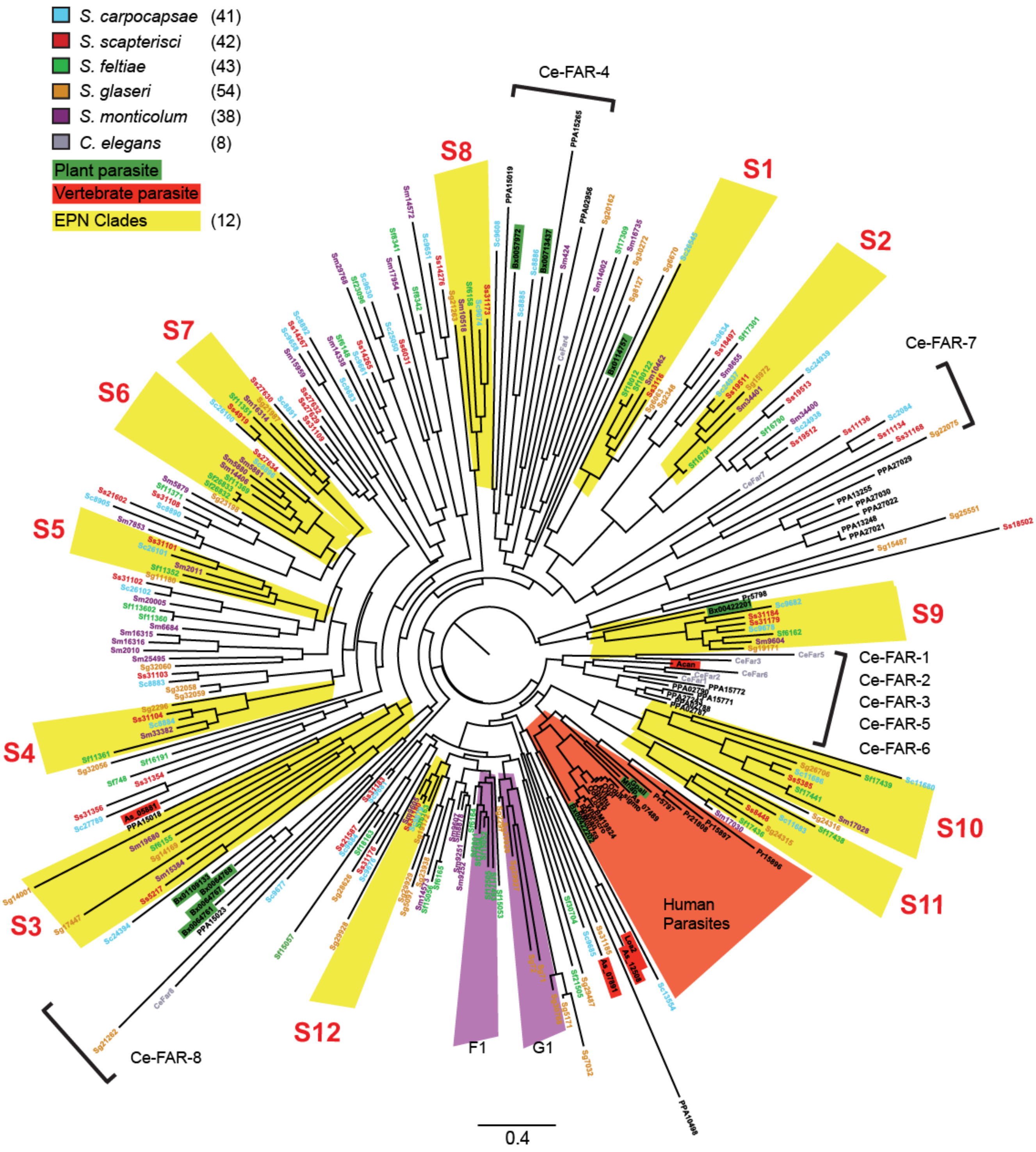

Figure S11. Protein neighbor-joining tree of fatty acid- and retinol-binding proteins among nematodes. Highlighted in large red numbers are the gene clades wherein each of the five *Steinernema* have at least one protein. This tree indicates that there are at least twelve monophyletic *Steinernema* FAR gene clades, illustrating the evolution and diversity of this gene family within *Steinernema*.

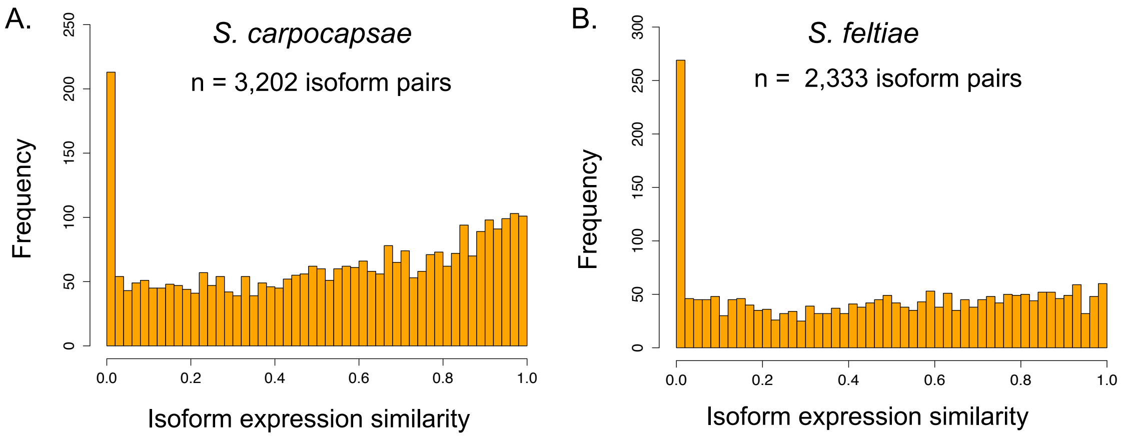

C.

Isoform expression similarity	No. of *S. carpocapsae* isoform pairs (3,202)	No. of *S. feltiae* isoform pairs (2,333)
> 0.95	248	126
< 0.50	1,377	1,189

Figure S12. Isoform expression similarity in *S. carpocapsae* and *S. feltiae*. Isoform expression similarity distribution of **A)** 3,202 isoform pairs in *S. carpocapsae*, and **B)** 2,333 isoform pairs in *S. feltiae* during development. Expression levels (in TPM) during development for isoforms of each gene were pairwise compared and the cosine similarity for each pair was calculated. Isoforms that had summed expression levels < 1 TPM during development were removed from the analysis. **C)** Table showing the number of isoform pairs that have similar or divergent expression profiles during development, that is, they have an isoform expression similarity > 0.95 or <0.5, respectively.

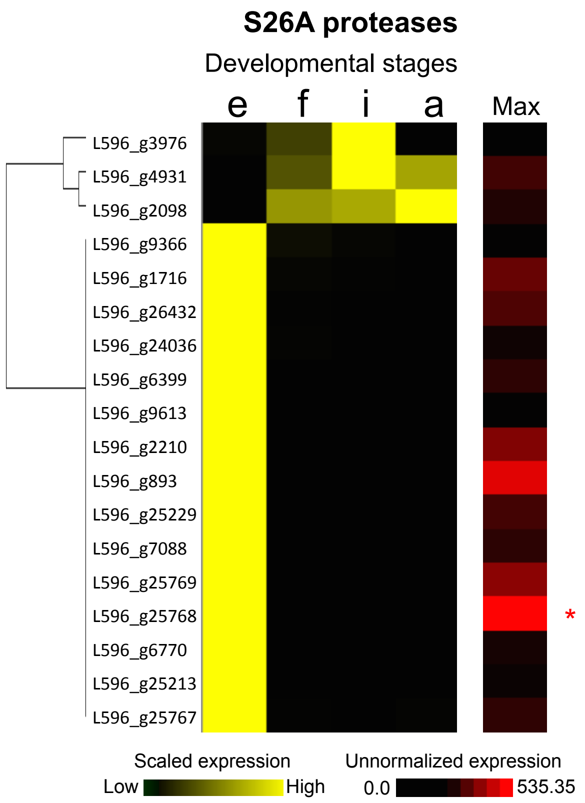

Figure S13. Stage-specific gene expression of S26 proteases in *S. carpocapsae*. Heat map of the scaled gene expression of 19 S26 proteases. Gene expression was scaled between 0 (minimum expression) and 1 (maximum expression), and hierarchically clustered based on the expression profiles. The FPKM column on the far right shows the maximum unnormalized gene expression values (FPKM) of each gene to show which genes are the most highly (red) or lowly expressed (black). The asterisk indicates the gene with the highest expression.

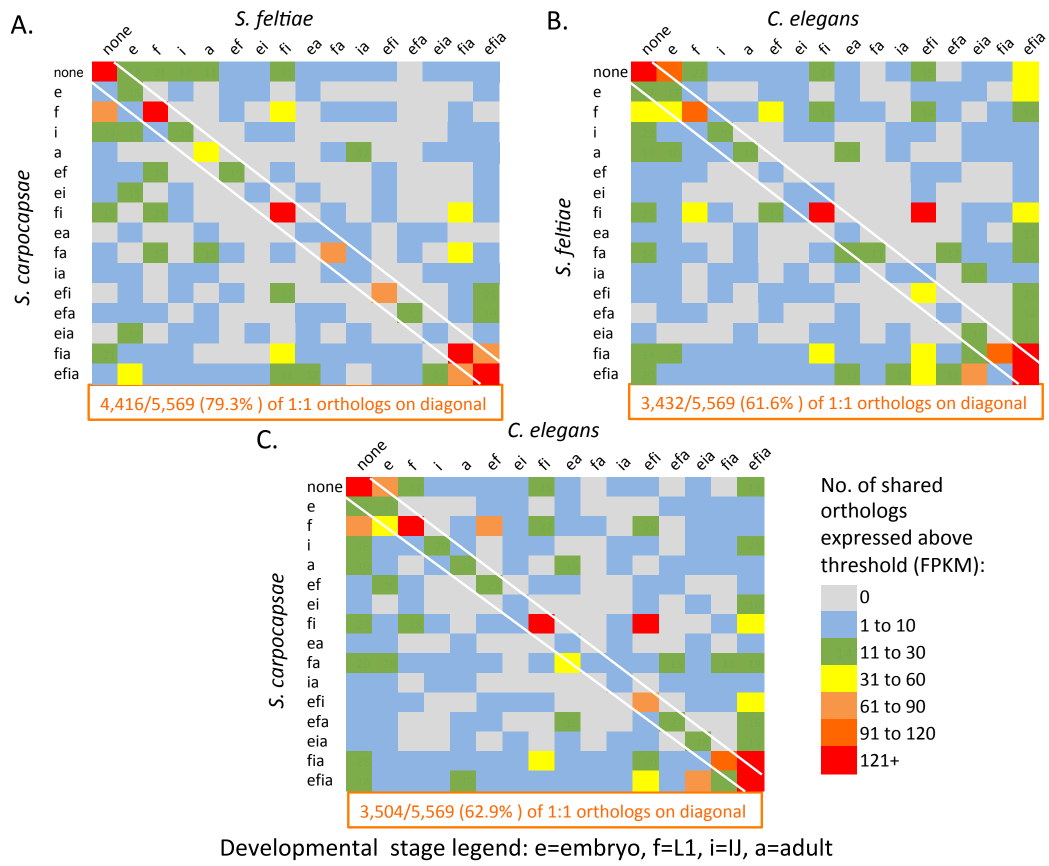

Figure S14. Gene expression conservation across closely and more distantly related nematode species. Gene expression conservation matrices showing the conserve developmental stage expression of 5,569 1:1:1 orthologs between **A)** *S. carpocapsae* and *C. elegans*, **B)** *S. feltiae* and *C. elegans*, and **C)** *S. carpocapsae* and *S. feltiae*. Gene orthologs that appear along the matrix diagonal are expressed above 10 FPKM in one species, and above at least 5 FPKM in the other species, in the same set of developmental stages, indicating that those orthologs have conserved stage-specific gene expression. Off-diagonal orthologs indicate that gene expression patterns of the orthologs have changed between the species being compared. Numbers of conserved orthologs and the percentages of orthologs that are conserved in stage-specific gene expression are listed below each matrix.

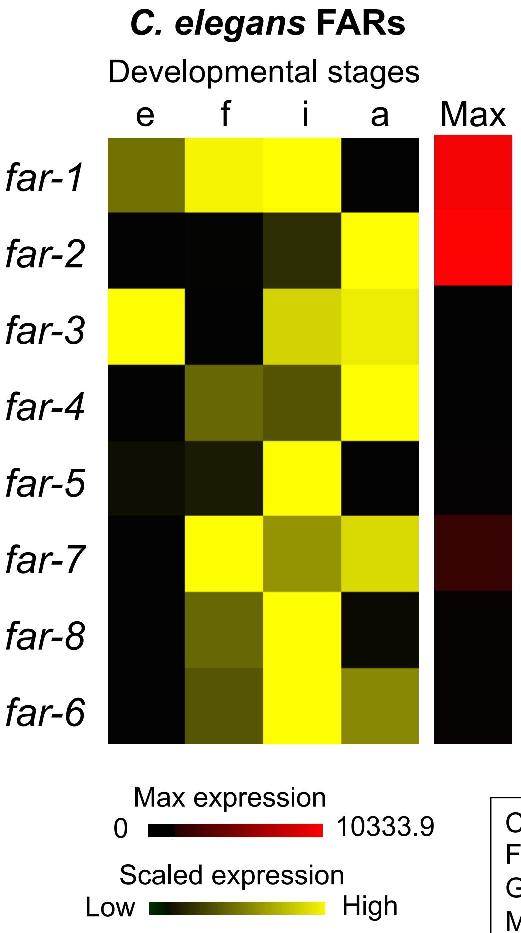

Orthologs in *Steinernema*	Orthologs in other nematodes	
C, F, G, M, S	B, R, A, HB	
---	B, R, J, A, HB	*
---	B, R, J, A	
---	B, R	
---	B, R, J	
---	B, R, J, A, HB	
---	B, R, J	
---	---	

Steinernema	Other nematodes
C = *S. carpocapsae*	B = *C. briggsae*
F = *S. feltiae*	R = *C. remanei*
G = *S. glaseri*	J = *C. japonica*
M = *S. monticolum*	A = *C. angaria*
S = *S. scapterisci*	HB = *H. bacteriophora*

Figure S15. *C. elegans* FAR expression and FAR conservation in other nematodes. Heat map of the scaled gene expression of 8 FAR proteins in *C. elegans*. Expression of each gene was scaled between 0 (minimum expression - black) and 1 (maximum expression - yellow) across the developmental stages. "e", "f", "i", and "a" correspond to the embryonic, first larval, infective juvenile, and young adult stages, respectively. The "Max" column shows each gene's maximum unnormalized expression value (FPKM) that was achieved during the time course. Genes that have high expression are in red, while genes that have low expression are in black. The asterisk indicates the gene that has the highest expression. The table to the right shows the FAR orthologs in *Steinernema* and in other sequenced nematode species. Only 1 FAR (*far-1*) in *C. elegans* is conserved in *Steinernema*,

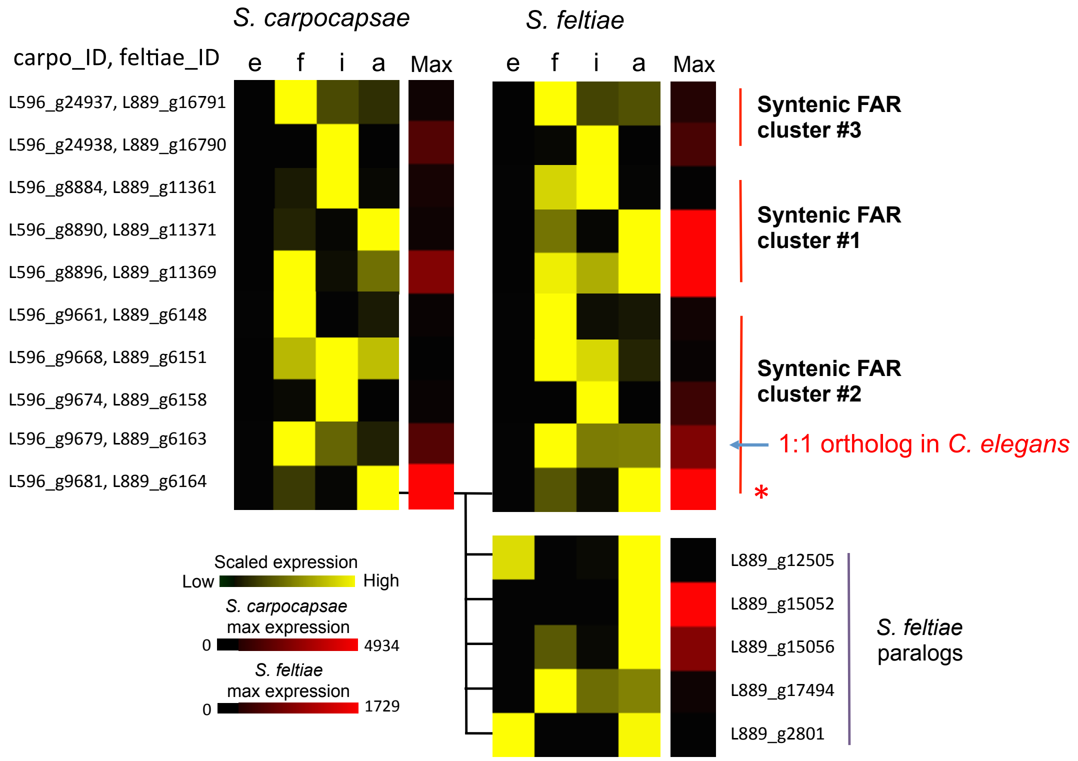

Figure S16. Gene expression of 10 1:1 orthologous FAR genes that are syntenic across three syntenic FAR clusters in *S. carpocapsae* and *S. feltiae*. For each FAR ortholog, gene expression was scaled between 0 (minimum expression) and 1 (maximum expression) across the developmental stages. The "Max" column shows the maximum gene expression levels (in FPKM) of the FARs in *S. carpocapsae* and *S. feltiae*. The asterisk indicates the gene that has the highest expression. Five *S. feltiae* paralogs of the bottommost ortholog in FAR syntenic cluster #2 are shown separately beneath the heat map. *S. carpocapsae* and *S. feltiae* gene IDs are shown next to the heat maps, as well as the only *S. carpocapsae* and *S. feltiae* ortholog that is conserved in *C. elegans* (*far-1*).

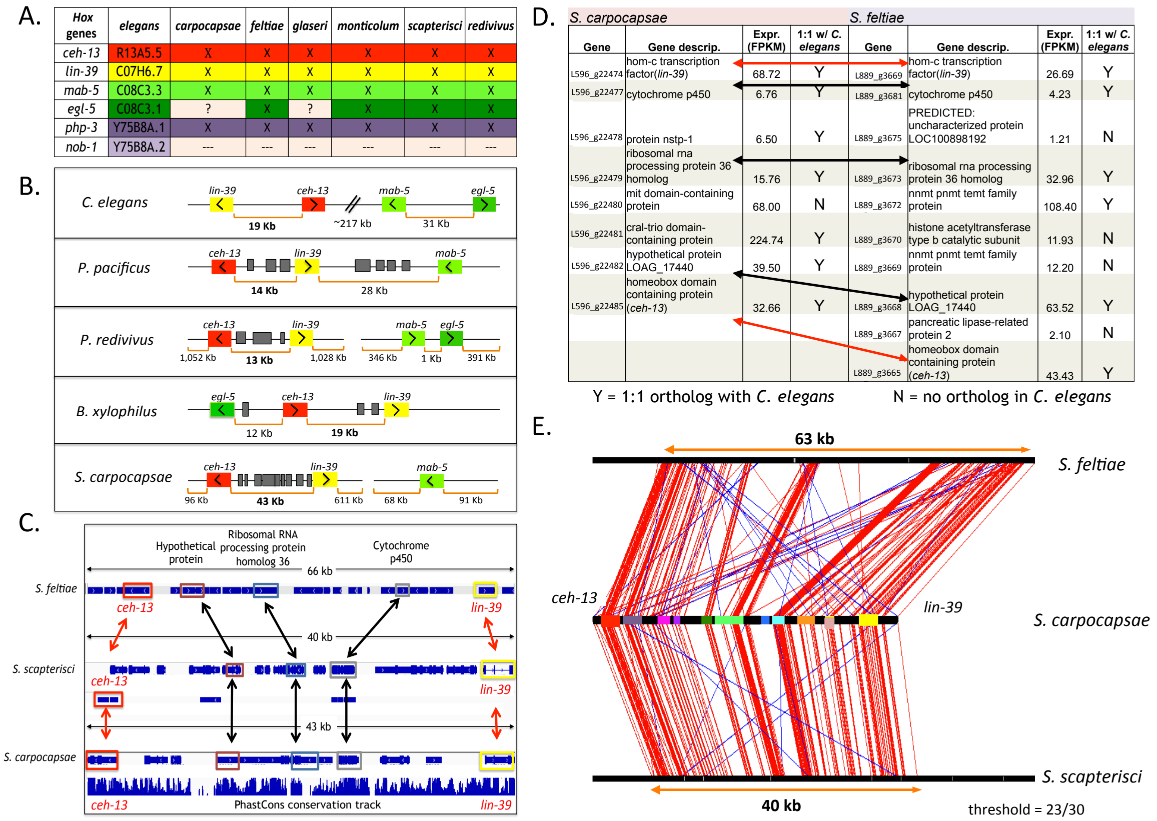

Figure S17. Hox cluster architecture in *Steinernema*. A) Table showing the presence of Hox genes in the genome assemblies of five *Steinernema* species, *C. elegans*, and *P. redivivus* (a close outgroup to *Steinernema*). "X" indicates the presence of the ortholog, "---" indicates the absence of the ortholog, and "?" indicates that the absence of the ortholog is likely due to incomplete assembly and annotation of that region. **B)** Hox cluster organization in four other sequenced nematode species and *S. carpocapsae*. *ceh-13* and *lin-39* are in close proximity to each other in every species, although species-specific reversals and insertions have occurred. **C)** Portion of the Hox gene cluster in *S. carpocapsae, S. scapterisci*, and *S. feltiae*. Black arrows indicate intervening genes that are present between *ceh-13* and *lin-39* across all three species. Red arrows indicate conserved Hox genes. **D)** Table of functional identities, expression levels, and sequence conservation of the genes embedded between the Hox genes *ceh-13* and *lin-39*. **E)** Alignment of the *ceh-13*/*lin-39* portion of the Hox cluster in *S. carpocapsae, S. feltiae*, and *S. scapterisci* using MUSSA [93]. Each colored line connecting the sequences indicates that 23/30 nts are conserved between their sequences. Red lines indicate that the sequences match in the same direction, whereas blue lines indicate reverse-complement matches.

A.

	S. carpocapsae FAR syntenic cluster #1 (8 FARs)	*S. carpocapsae* FAR syntenic cluster #2 (11 FARs)	*S. carpocapsae* FAR syntenic cluster #3 (3 FARs)
S. scapterisci	5	5	3
S. feltiae	3	5	2
S. glaseri	0	0	0
S. monticolum	0	0	2

B.

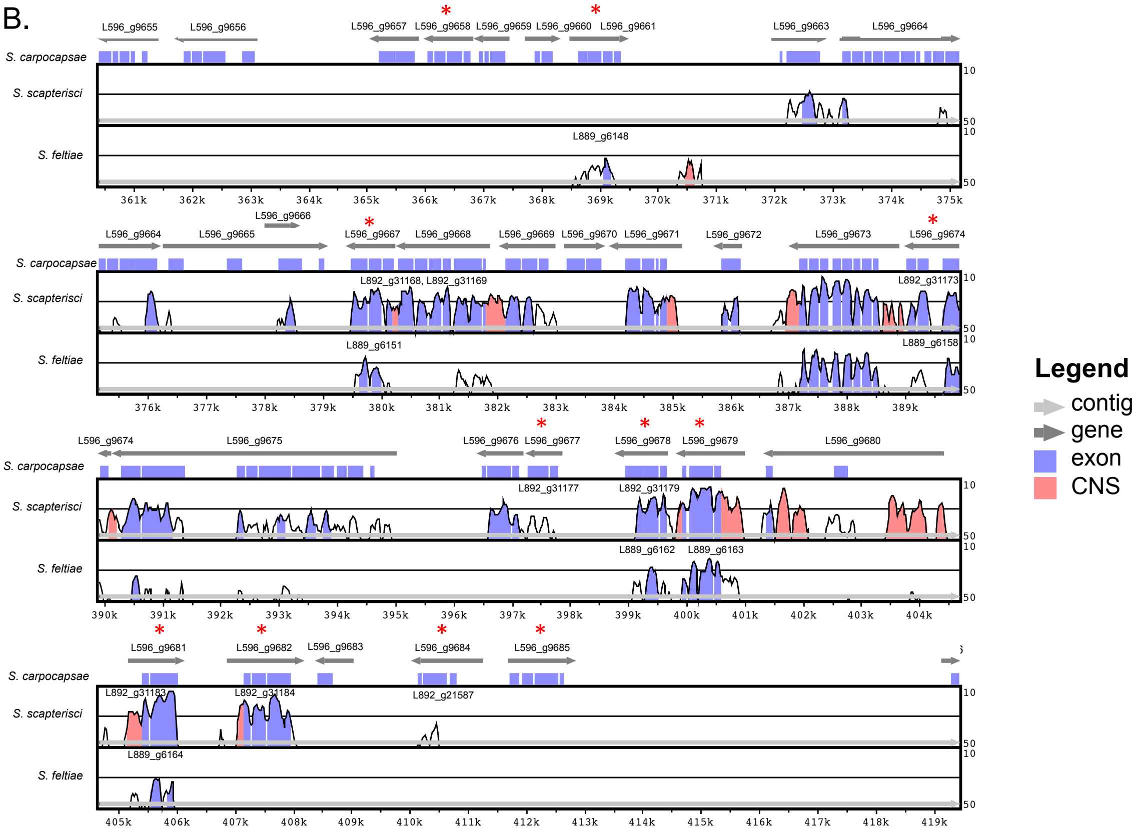

Figure S18. FAR synteny in *Steinernema*. A) Numbers of 1:1 FAR orthologs that are syntenic in *S. carpocapsae* and the other *Steinernema* species. **B)** Multiple sequence alignment of a syntenic cluster (FAR cluster #2) of fatty acid- and retinol- binding (FAR) proteins from *S. carpocapsae*, *S. feltiae*, and *S. scapterisci.* Red asterisks denote FAR genes in *S. carpocapsae*. Peaks show DNA sequences that are conserved across species. Lavender regions indicate exons, while peach colored regions indicate conserved non-coding sequences.

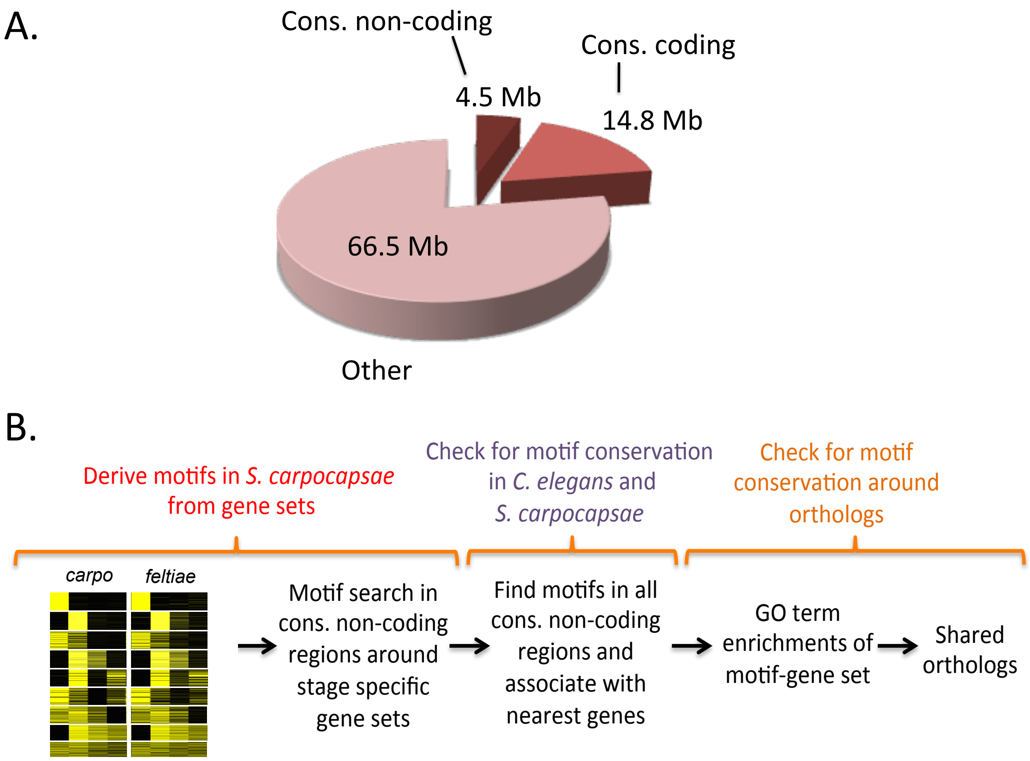

Figure S19. Using *Steinernema* genome conservation to find conserved regulatory motifs across species. A) Pie chart showing the fraction of the 86 Mb *S. carpocapsae* genome that comprises conserved coding, non-coding, and other sequence. **B)** General workflow for the motif analysis.

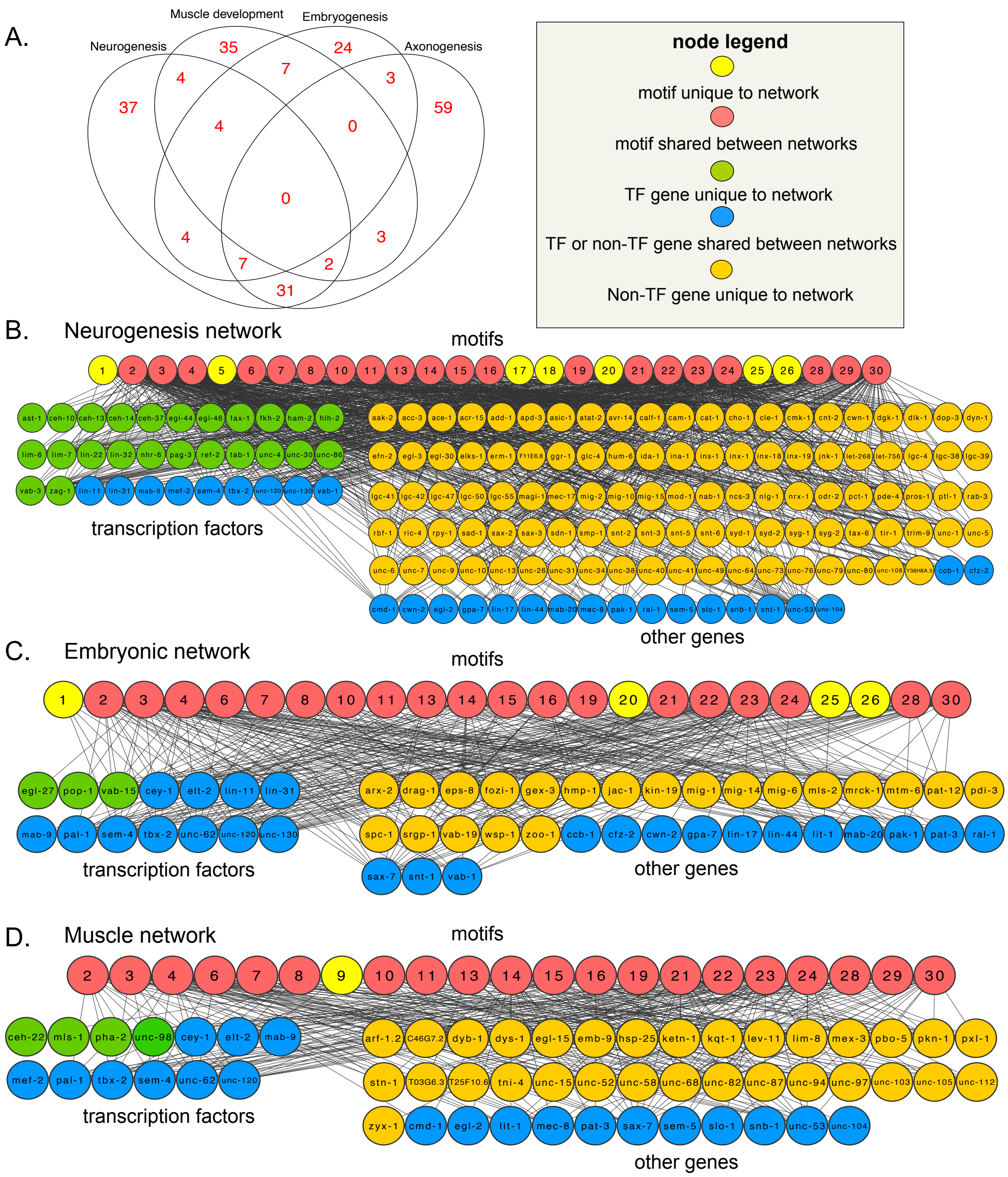

Figure S20. Motif and target gene associations that are conserved between *C. elegans* and *S. carpocapsae*. A) A venn diagram showing the number of motif-associated genes that are shared across four GO term networks (neurogenesis, axonogenesis, muscle development, and embryogenesis). **B-D)** Networks showing all conserved *S. carpocapsae* and *C. elegans* motif-target gene associations related to sets of neurogenesis/axonogenesis, embryogenesis, and muscle GO terms. Nodes for motifs and genes that are shared by any of the three networks are highlighted in yellow and blue, respectively.

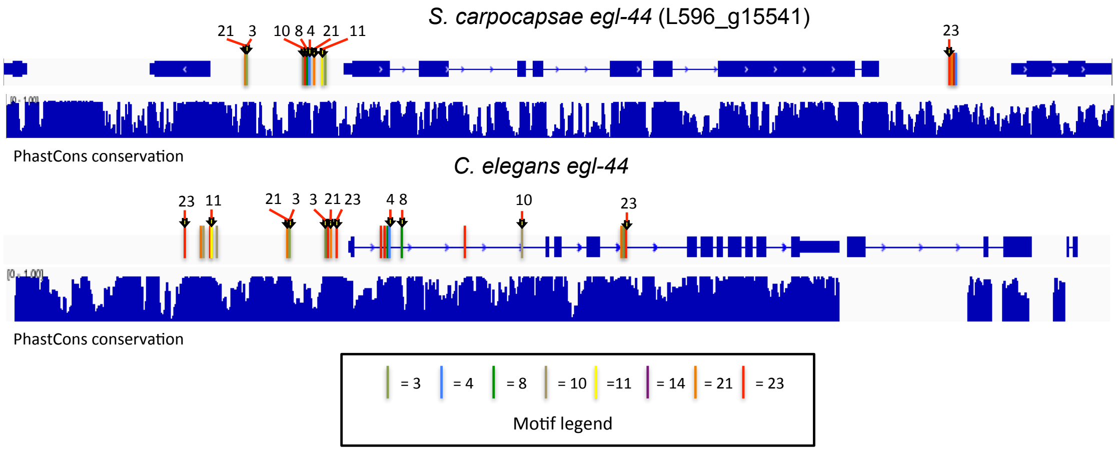

Figure S21. Conservation of motif location around orthologous genes. *egl-44* gene model in *S. carpocapsae* and *C. elegans* showing conserved motifs. Sequence conservation tracks are displayed below each gene model.
