# Supplementary material for: Comparative genomics of Steinernema reveals deeply conserved gene regulatory networks
Source: Genome Biol. 2015 Sep 21;16:200. doi: 10.1186/s13059-015-0746-6 (PMC4578762; doi:10.1186/s13059-015-0746-6)
Supplement: Additional file 2: — Supplementary tables and legends. (PDF 1760 kb) [file 13059_2015_746_MOESM2_ESM.pdf]

Species	Total proteins in analysis	Proteins clustered	Orphan proteins	% orphans
S. carpocapsae	28,313	17,653	10,660	37.89
B. xylophilus	18,074	12,420	5,654	31.28
P. redivivus	24,249	17,527	6,722	27.72
C. elegans	20,426	15,930	4,496	22.01
P. pacificus	24,217	15,042	9,175	37.89
A. suum	18,842	10,926	7,616	41.07
B. malayi	21,332	16,066	5,266	24.69
T. spiralis	16,380	11,066	5,314	32.44
M. hapla	13,072	8,889	4,183	32.00
N. vitripennis	18,822	15,112	3,710	19.71

Table S1. Cluster analysis of orphan proteins.

Species	# of CEGMA protein orthologs	% of CEGMA orthologs in assembly
S. carpocapsae	445	98.02%
S. feltiae	443	97.58%
S. glaseri	440	96.92%
S. monticolum	437	96.26%
S. scapterisci	441	97.14%

Table S2. Predicted *Steinernema* genome completeness using CEGMA.

	No. of *S. carpocapsae* transcripts (31,944)	No. of *S. scapterici* transcripts (33,149)	No. of *S. feltiae* transcripts (36,434)	No. of *S. glaseri* transcripts (37,120)	No. of *S. monticolum* transcripts (38,381)
Full support	10,408	4,338	5,971	4,499	5,708
Partial support	15,297	15,811	16,184	14,783	14,359
No support	6,239	13,000	14,279	17,838	18,314

	No. of *S. carpocapsae* transcripts (31,944)	No. of *S. scapterici* transcripts (33,149)	No. of *S. feltiae* transcripts (36,434)	No. of *S. glaseri* transcripts (37,120)	No. of *S. monticolum* transcripts (38,381)
Full support	32.6%	13.0%	16.4%	12.1%	14.9%
Partial support	47.9%	47.7%	44.4%	39.8%	37.4%
No support	19.5%	39.2%	39.2%	48.1%	47.7%

Table S3. Number and percentage of *S. carpocapsae*, *S.* scapterisci, *S. feltiae*, *S. glaseri*, and *S. monticolum* transcripts that are fully, partially, or not supported by RNA-seq reads. Four RNA-seq data sets (embryo, L1, IJ, and adult) contribute to the transcript support for *S. carpocapsae*, while only single IJ RNA-seq data sets contribute to the transcript support for all the other *Steinernema* species.

Type of Proteases	Scar	Ssca	Sfel	Sgla	Smon	Bxyl	Pred	Cele	Ppac
Aspartic	41	66	40	39	67	78	24	27	38
Cysteine	124	147	147	116	122	148	112	141	100
Metallo	214	268	306	314	307	209	232	203	215
Serine	246	347	301	253	349	174	227	156	237
Threonine	27	36	34	29	32	21	21	23	22
Unassigned	2	7	4	5	4	1	1	1	1
Total Proteases	654	871	831	756	881	631	617	551	613
Total Protease inhibitors	146	175	214	133	153	60	91	94	64

Table S4. Protease abundance summary.

Subdivisions	Scar	Ssca	Sfel	Sgla	Smon	Bxyl	Pred	Cele	Ppac
A1A (A01A)	34	60	32	34	56	72	19	22	32
A8	0	0	1	0	0	0	0	0	0
A11A	0	0	0	0	0	0	0	0	0
A11X	3	1	2	0	7	1	1	0	0
A22A	2	2	2	3	2	3	2	3	4
A22B	2	3	2	2	2	2	2	2	2
A24A	0	0	1	0	0	0	0	0	0
Total aspartic proteases	41	66	40	39	67	78	24	27	38

Table S5. Aspartic protease abundance.

Subdivisions	Scar	Ssca	Sfel	Sgla	Smon	Bxyl	Pred	Cele	Ppac
C1A (C01A)	46	53	57	18	37	66	38	31	27
C2A	6	6	6	7	6	6	6	13	5
C11	0	0	0	0	0	0	0	0	0
C12	2	1	3	3	4	3	0	4	2
C13	4	4	7	6	8	16	6	2	3
C14A	1	5	1	3	1	2	2	4	1
C14B	1	1	1	1	1	1	1	1	1
C15	1	2	1	0	1	1	1	3	0
C19	22	24	27	28	21	22	23	38	24
C26	3	5	3	6	3	2	2	2	2
C39	0	0	0	0	1	0	0	0	0
C40	0	0	0	1	1	0	0	0	0
C44	7	8	7	11	9	8	5	8	5
C46	8	8	7	5	5	3	6	10	10
C48	4	4	4	6	3	5	4	4	5
C50	2	3	2	4	4	1	1	2	0
C54	2	2	3	2	2	2	4	2	1
C56	3	5	2	3	4	2	2	3	1
C64	1	2	3	2	2	2	1	1	1
C65	1	2	2	1	1	1	1	1	1
C67	1	0	2	1	1	0	0	1	0
C74	1	1	2	0	1	0	1	1	0
C78	1	1	1	2	1	1	1	1	1
C80	0	1	0	0	0	0	0	0	0
C83	1	1	1	1	1	0	0	1	1
C85	1	3	1	1	1	1	2	2	0
C86	4	4	1	3	2	2	2	2	2
C89	1	1	2	1	1	1	3	3	7
Total cysteine proteases	124	147	146	116	122	148	148	141	100

Table S6. Cysteine protease abundance.

Subdivisions	Scar	Ssca	Sfel	Sgla	Smon	Bxyl	Pred	Cele	Ppac
T01A	19	22	21	16	16	14	11	14	12
T01B	0	0	0	1	0	0	0	0	0
T01X	0	0	0	0	0	0	1	0	0
T02	2	2	2	1	3	2	2	3	1
T03	6	10	11	9	12	5	7	6	9
T05	0	0	0	2	1	0	0	0	0
T06	0	2	0	0	0	0	0	0	0
Total threonine proteases	27	36	34	29	32	21	21	23	22

Table S7. Threonine protease abundance.

Subdivisions	Scar	Ssca	Sfel	Sgla	Smon	Bxyl	Pred	Cele	Ppac
M01	27	24	26	16	20	26	27	15	17
M02	1	1	1	1	2	1	1	0	1
M03A	2	7	3	3	4	3	3	2	4
M08	1	1	2	2	1	2	1	0	1
M10A	9	8	8	15	21	6	7	7	6
M12A	37	54	103	106	78	25	37	39	53
M12B	10	10	11	12	9	10	11	11	14
M13	20	21	22	23	26	64	65	31	28
M14A	26	23	19	16	24	10	12	9	9
M14B	4	4	6	3	4	4	5	4	4
M14X	1	1	1	2	1	1	1	1	0
M15A	0	0	0	0	0	0	0	0	0
M15B	0	0	0	0	0	0	0	0	0
M15C	0	0	0	0	0	0	0	0	0
M15D	0	0	0	0	0	0	0	0	0
M16A	16	32	12	21	3	0	0	15	3
M16B	4	5	9	6	10	7	10	11	13
M16C	2	3	3	3	3	2	2	1	1
M17	2	3	3	4	4	2	2	2	5
M18	1	1	2	1	2	1	2	1	1
M19	0	0	0	0	2	0	0	0	0
M20A	5	6	10	8	11	7	4	7	7
M20B	0	0	0	0	1	0	0	0	0
M20C	0	1	0	0	1	0	0	0	0
M20D	0	1	0	1	3	0	0	0	0
M20X	0	0	0	1	0	0	0	0	0
M22	2	6	4	4	3	3	2	3	2
M23B	2	7	1	5	4	0	1	4	1
Total table S8 metalloproteases	172	219	246	253	236	174	193	163	170

Table S8. Metallo-protease abundance part I.

Subdivisions	Scar	Ssca	Sfel	Sgla	Smon	Bxyl	Pred	Cele	Ppac
M24A	2	2	3	4	6	2	3	4	2
M24B	5	6	7	7	8	4	4	4	5
M24X	2	2	5	3	4	2	2	2	3
M28A	1	1	2	1	2	1	0	1	0
M28B	6	6	9	5	8	3	6	3	4
M28E	0	0	0	0	0	0	0	0	0
M28X	3	3	4	3	3	2	6	3	3
M38	5	7	8	14	16	6	5	6	6
M41	4	4	4	4	4	4	3	6	2
M43B	0	0	0	0	0	0	1	0	0
M48A	1	1	1	1	1	1	1	1	3
M48B	0	0	0	1	0	0	0	0	0
M48X	0	3	1	4	2	0	0	0	0
M49	1	1	1	1	1	1	1	1	5
M50	0	0	0	0	0	0	0	0	0
M50A	1	1	2	0	1	2	2	2	2
M50B	0	0	0	2	0	0	0	0	0
M61	0	0	0	0	1	0	0	0	0
M67A	7	6	6	8	8	5	4	5	8
M67C	0	1	2	0	1	0	0	0	0
M67X	4	5	4	3	4	2	1	2	2
M75	0	0	1	0	1	0	0	0	0
Total table S8 metalloproteases	42	49	60	61	71	35	39	40	45
Total table S9 metalloproteases	172	219	246	253	236	174	193	163	170
Total metalloproteases	214	268	306	314	307	209	232	203	215

Table S9. Metallo-protease abundance part II.

Subdivisions	Scar	Ssca	Sfel	Sgla	Smon	Bxyl	Pred	Cele	Ppac
S01A	82	127	98	94	119	4	57	5	26
S01B	0	0	0	1	1	0	0	0	0
S01D	0	0	0	0	0	0	0	0	0
S01X	1	1	2	1	1	1	2	2	2
S08A	3	4	1	3	4	6	12	1	2
S08B	4	4	5	8	5	3	7	4	4
S08X	0	0	0	0	1	0	0	0	0
S09A	2	6	3	4	4	1	2	0	2
S09B	2	2	2	2	6	2	4	3	4
S09C	3	2	2	2	4	5	2	2	4
S09D	0	0	0	0	0	0	0	0	0
S09X	60	66	59	47	53	63	69	61	71
S10	21	27	16	19	26	29	18	21	65
S11	2	1	0	0	2	0	0	0	0
S12	9	10	13	8	15	6	8	9	8
S13	0	1	0	2	2	0	0	0	0
S14	1	5	1	2	3	1	1	1	1
S15	0	0	0	0	1	0	0	0	0
S16	2	3	39	8	30	2	3	9	1
S24	0	5	1	2	1	0	0	0	0
S26A	18	43	12	4	9	2	2	2	1
S26B	1	1	1	1	1	1	1	1	2
S26C	0	0	0	0	1	0	0	0	0
S28	16	16	13	13	29	25	20	15	20
S33	12	14	18	17	16	18	12	14	15
S41A	0	0	0	2	4	0	0	0	0
S45	0	0	0	2	0	0	0	0	0
S46	0	0	0	0	1	0	0	0	0
Total table S10 serine proteases	239	338	286	242	339	169	220	150	228

Table S10. Serine protease abundance part I.

Subdivisions	Scar	Ssca	Sfel	Sgla	Smon	Bxyl	Pred	Cele	Ppac
S49	0	1	2	0	2	0	0	0	0
S54	6	6	8	7	5	3	5	4	7
S58	0	0	0	0	0	0	0	0	0
S59	1	1	1	2	1	1	1	1	1
S63	0	0	4	1	2	1	1	0	1
S66	0	0	0	1	0	0	0	0	0
S72	0	0	0	0	0	0	0	1	0
S73	0	1	0	0	0	0	0	0	0
Total table S10 serine proteases	7	9	15	11	10	5	7	6	9
Total table S11 serine proteases	239	338	286	242	339	169	220	150	228
Total serine proteases	246	347	301	253	349	174	227	156	237

Table S11. Serine protease abundance part II.

Subdivisions	Scar	Ssca	Sfel	Sgla	Smon	Bxyl	Pred	Cele	Ppac
I01	1	1	2	0	1	0	1	1	0
I02	30	27	42	36	30	40	48	49	24
I04	18	34	63	16	39	3	4	7	9
I08	37	40	49	38	30	3	20	14	12
I11	0	1	0	0	0	0	0	0	0
I21	1	1	2	1	1	1	1	1	1
I25B	7	7	7	6	7	3	3	3	4
I29	3	1	2	1	0	1	2	2	0
I31	2	2	2	1	2	2	0	1	1
I32	1	1	1	1	0	1	2	2	1
I33	0	0	0	0	0	1	0	1	0
I35	1	1	0	0	2	0	0	1	2
I39	2	1	2	3	2	3	2	2	1
I51	7	5	3	5	3	2	2	2	4
I63	36	53	39	25	36	0	6	8	5
Total protease inhibitors	146	175	214	133	153	60	91	94	64

Table S12. Protease inhibitor abundance.

	Scar	Ssca	Sfel	Sgla	Smon	Bxyl	Pred	Cele	Ppac
FAR proteins	41	42	43	54	38	9	5	8	19
FAR proteins with signal peptide	31	32	30	27	30	9	4	7	16

	Asum	Tspi	Bmal
FAR proteins	4	0	3

Table S13. Fatty acid- and retinoid-binding protein abundance in *Steinernema* species.

GEO accession	Species	DNA or RNA	Developmental stage	Stranded or unstranded	Paired or single end reads	Read length (bp)	Sequencer	Trimmed read length (bp)	Insert length (bp)	No. of reads (Million)
SRX1019157	*S. carpocapsae*	RNA	early embryo rep 1	first-strand	single	75	NextSeq 500	35	none	27.14
SRX1019158	*S. carpocapsae*	RNA	early embryo rep 2	first-strand	single	75	NextSeq 500	35	none	19.13
SRX1019159	*S. carpocapsae*	RNA	L1 rep 1	first-strand	single	50	Illumina HiSeq 2000	35	none	37.77
SRX1019160	*S. carpocapsae*	RNA	L1 rep 2	first-strand	single	50	Illumina HiSeq 2000	35	none	39.67
SRX1019161	*S. carpocapsae*	RNA	IJ rep 1	first-strand	single	50	Illumina HiSeq 2000	35	none	31.04
SRX1019162	*S. carpocapsae*	RNA	IJ rep 2	first-strand	single	50	Illumina HiSeq 2000	35	none	31.75
SRX1019163	*S. carpocapsae*	RNA	young adult rep 1	first-strand	single	50	Illumina HiSeq 2000	35	none	27.60
SRX1019164	*S. carpocapsae*	RNA	young adult rep 2	first-strand	single	50	Illumina HiSeq 2000	35	none	28.41

GEO accession	Species	DNA or RNA	Developmental stage	Stranded or unstranded	Paired or single end reads	Read length (bp)	Sequencer	Trimmed read length (bp)	Insert length (bp)	No. of reads (Million)
SRX1019165	*S. feltiae*	RNA	early embryo rep 1	first-strand	single	75	NextSeq 500	35	none	16.85
SRX1019166	*S. feltiae*	RNA	early embryo rep 2	first-strand	single	75	NextSeq 500	35	none	23.43
SRX1019168	*S. feltiae*	RNA	L1 rep 1	first-strand	single	75	NextSeq 500	35	none	21.76
SRX1019169	*S. feltiae*	RNA	L1 rep 2	first-strand	single	75	NextSeq 500	35	none	23.13
SRX1019170	*S. feltiae*	RNA	IJ rep 1	first-strand	single	75	NextSeq 500	35	none	29.62
SRX1019171	*S. feltiae*	RNA	IJ rep 2	first-strand	single	75	NextSeq 500	35	none	20.17
SRX1019172	*S. feltiae*	RNA	young adult rep 1	first-strand	single	75	NextSeq 500	35	none	20.83
SRX1019173	*S. feltiae*	RNA	young adult rep 2	first-strand	single	75	NextSeq 500	35	none	22.94

GEO accession	Species	DNA or RNA	Developmental stage	Stranded or unstranded	Paired or single end reads	Read length (bp)	Sequencer	Trimmed read length (bp)	Insert length (bp)	No. of reads (Million)
SRX1019174	*C. elegans*	RNA	embryo rep 1	first-strand	single	75	NextSeq 500	35	none	22.79
SRX1019175	*C. elegans*	RNA	embryo rep 2	first-strand	single	75	NextSeq 500	35	none	24.31
SRX1019176	*C. elegans*	RNA	L1 rep 1	first-strand	single	75	NextSeq 500	35	none	20.94
SRX1019177	*C. elegans*	RNA	L1 rep 2	first-strand	single	75	NextSeq 500	35	none	35.13
SRX1019178	*C. elegans*	RNA	dauer rep 1	first-strand	single	75	NextSeq 500	35	none	42.04
SRX1019179	*C. elegans*	RNA	dauer rep 2	first-strand	single	75	NextSeq 500	35	none	40.20
SRX1019180	*C. elegans*	RNA	adult rep 1	first-strand	single	75	NextSeq 500	35	none	44.73
SRX1019181	*C. elegans*	RNA	adult rep 2	first-strand	single	75	NextSeq 500	35	none	37.89

Table S14. RNA library statistics for gene expression analysis.

	Total # 1:1 orthologs	Sum of 1:1s that are syntenic across scaffolds that have 2+ 1:1s	Sum of 1:1s that are syntenic across scaffolds that have 10+ 1:1s
S. scapterisci – S. carpocapsae	12,395	11,272	6,576
S. feltiae – S. carpocapsae	10,228	8,188	4,475
S. monticolum – S. carpocapsae	10,129	6,179	331
S. glaseri – S. carpocapsae	9,731	7,433	2,337

Table S15. Syntenic one-to-one orthologous genes in *Steinernema*.

	Max # of syntenic 1:1s on a scaffold	*S. carpo* scaffold ID with the most 1:1s shared with another *Steinernema* scaffold	*Steinernema* scaffold ID with the most 1:1s shared with a *S. carpo* scaffold	Total length of *S. carpo* scaffold with most 1:1s (kb)	Total length of *Steinernema* scaffold with most 1:1s (kb)	Distance between outermost 1:1s on *S. carpo* scaffold (kb)	Distance between outermost 1:1s on *Steinernema* scaffold (kb)	No. of non-syntenic/ non-1:1s between outermost syntenic 1:1s in *S. carpo*	No. of non-syntenic/ non-1:1s between outermost syntenic 1:1s in *Steinernema*
S. scapterisci – S. carpocapsae	103	1096	2381	994	453	374	397	38	51
S. feltiae – S. carpocapsae	191	1189	5455	915	1,446	878	794	89	82
S. monticolum – S. carpocapsae	20	1409	8466	1,136	66	108	54	18	5
S. glaseri – S. carpocapsae	44	1250	6476	909	288	209	245	17	49

Table S16. Synteny statistics for *Steinernema* scaffolds with most syntenic orthologous genes.

Motif No.	Web Logo	No. of *S. carpocapsae* gene neighbors	No. of *C. elegans* gene neighbors	
1	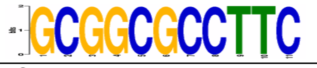	1,882	1,274	*
2	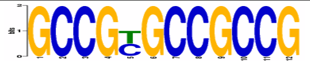	2,662	4,879	*
3	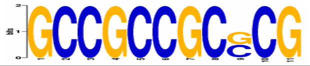	3,156	4,594	*
4	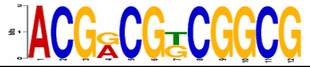	3,831	2,527	*
5	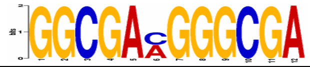	944	769	
6	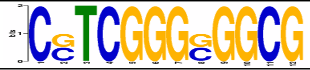	556	2,120	
7	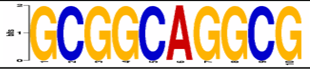	1,412	680	*
8	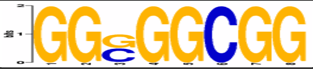	989	5,114	
9	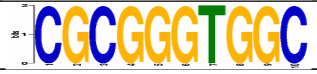	426	677	*

Motif No.	Web Logo	No. of *S. carpocapsae* gene neighbors	No. of *C. elegans* gene neighbors	
10	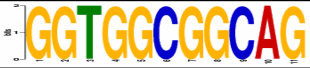	1,755	5,461	*
11	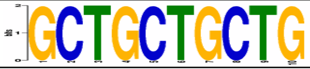	1,033	810	*
12	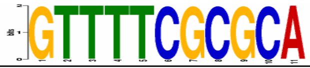	1,531	19	*
13	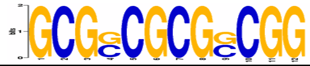	1,277	2,931	*
14	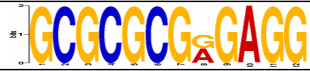	3,147	4,437	*
15	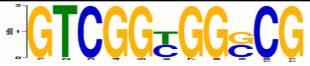	2,111	2,525	*
16	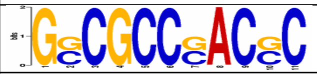	994	3,861	
17	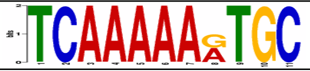	613	2,446	*
18	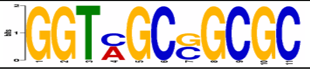	334	1,394	*

Motif No.	Web Logo	No. of *S. carpocapsae* gene neighbors	No. of *C. elegans* gene neighbors	
19	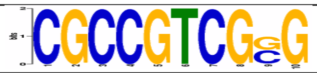	2,444	1,295	*
20	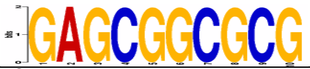	1,273	839	*
21	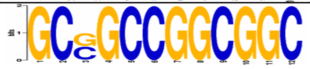	4,026	2,929	*
22	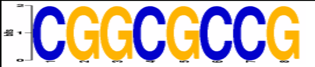	2,235	1,256	
23	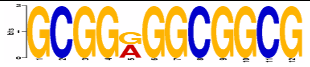	4,853	6,729	*
24	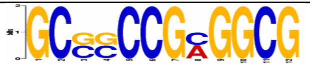	3,306	3,885	*
25	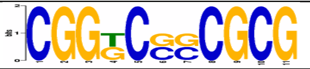	757	2,481	*
26	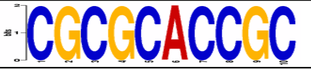	1,339	784	*
27	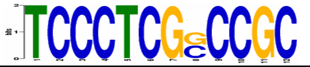	402	4,230	*

Motif No.	Web Logo	No. of *S. carpocapsae* gene neighbors	No. of *C. elegans* gene neighbors	
28	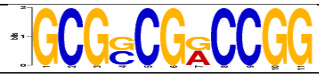	1,938	1,676	
29	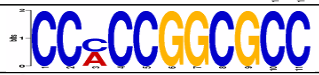	487	4,790	*
30	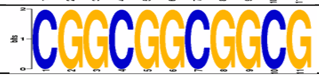	4,805	3,383	*

Table S17. Putative regulatory motifs discovered. The final set of putative regulatory motifs, their web logo sequences, and the number *S. carpocapsae* and *C. elegans* genes neighboring each motif. A red asterisk indicates that the motif matches one of more WormBase motifs (p-value < $1e^{-4}$ and e-value < 0.5).

Motif No.	Motif Match (p-value < $1e^{-4}$ and e-value < 0.5)
1	Y47D9A.1b.6_Stormo_2012, T06C10.2.8_Stormo_2012
2	M03F4.3a.4_Stormo_2012, Elemento_2005_motif_239, C36C9.4.3_Stormo_2012
3	Y46E12BL.4.5_Stormo_2012
4	Y40B10A.8.2_Stormo_2012, C04F2.1.3_Stormo_2012
5	No match
6	No match
7	C48E7.5.1_Stormo_2012, B0205.4.9_Stormo_2012, C54D2.4b.9_Stormo_2012
8	No match
9	W06H12.1.1_Stormo_2012, C53B7.5.7_Stormo_2012, Elemento_2005_motif_272

Motif No.	Motif Match (p-value < $1e^{-4}$ and e-value < 0.5)
10	C23H5.1.3_Stormo_2012, F09F7.7b.7_Stormo_2012
11	Elemento_2005_motif_260, Y44E3A.2.1_Stormo_2012, Elemento_2005_motif_151, F19H6.1.2_Stormo_2012, F36H9.4.7_Stormo_2012, K10B4.1.1_Stormo_2012, Elemento_2005_motif_56
12	C26D10.1.9_Stormo_2012, B0511.14b.3_Stormo_2012, C23H3.1.8_Stormo_2012
13	F33D11.7.1_Stormo_2012, M199.1.4_Stormo_2012, C49C8.5.1_Stormo_2012, F35H10.4.2_Stormo_2012, Y73E7A.1a.7_Stormo_2012, PAR2.3b.1_Stormo_2012, M03F4.3a.4_Stormo_2012
14	F56F11.1.9_Stormo_2012, F17A9.3.2_Stormo_2012, Elemento_2005_motif_111, R13H4.7.7_Stormo_2012
15	F34H10.2.6_Stormo_2012, C52B9.3b.7_Stormo_2012
16	No match

Motif No.	Motif Match (p-value < $1e^{-4}$ and e-value < 0.5)
17	Y71D11A.2b.7_Stormo_2012
18	ZC518.2.9_Stormo_2012
19	F22D6.10.2_Stormo_2012
20	T24C12.4.7_Stormo_2012
21	W03D2.9.1_Stormo_2012, R155.1a.8_Stormo_2012, B0205.4.9_Stormo_2012, F59F3.2.10_Stormo_2012
22	No match
23	Y49C4A.4.3_Stormo_2012, F55G1.1.7_Stormo_2012, C23H5.1.1_Stormo_2012
24	F42H10.3.3_Stormo_2012, F52F10.5.6_Stormo_2012, C49C8.5.1_Stormo_2012, W04C9.6.7_Stormo_2012, C40C9.2.10_Stormo_2012, R12A1.3.4_Stormo_2012

Motif No.	Motif Match (p-value < $1e^{-4}$ and e-value < 0.5)
25	F13D12.2.1_Stormo_2012
26	F17H10.2a.3_Stormo_2012, Elemento_2005_motif_348
27	K01C8.6.1_Stormo_2012
28	No match
29	F53B1.2.10_Stormo_2012, F31D5.3b.3_Stormo_2012, F35B3.5a.2_Stormo_2012, F35B3.5a.1_Stormo_2012
30	Y40B10A.8.2_Stormo_2012, Zhao_et.al_2007_3, C23H5.1.1_Stormo_2012, F52E1.9.2_Stormo_2012, C47D12.3.1_Stormo_2012, F58A4.3.9_Stormo_2012, C25D7.5.3_Stormo_2012, C04F2.1.3_Stormo_2012, PAR2.3a.9_Stormo_2012

Table S18. Putative regulatory motif matches to Wormbase motifs.

Network	GO ID	GO description
Neural	GO:0022008	neurogenesis
	GO:0048699	generation of neurons
	GO:0030182	neuron differentiation
	GO:0048666	neuron development
	GO:0007399	nervous system development
	GO:0045664	regulation of neuron differentiation
	GO:0014016	neuroblast differentiation
	GO:0050767	regulation of neurogenesis
	GO:0048667	cell morphogenesis involved in neuron differentiation
	GO:0048665	neuron fate specification
	GO:0050769	positive regulation of neurogenesis
	GO:0014017	neuroblast fate commitment
	GO:0045666	positive regulation of neuron differentiation
	GO:0007409	axonogenesis
	GO:0050770	regulation of axonogenesis
	GO:0007411	axon guidance
	GO:0061564	axon development
	GO:0033563	dorsal/ventral axon guidance
	GO: 0045773	positive regulation of axon extension

Network	GO ID	GO description
Embryo	GO:0045995	regulation of embryonic development
	GO:0048598	embryonic morphogenesis
	GO:0007369	gastrulation
	GO:0001704	formation of primary germ layer
	GO:0042074	cell migration involved in gastrulation
	GO:0060795	cell fate commitment involved in formation of primary germ layer
	GO:0016055	Wnt signaling pathway
	GO:0007509	mesoderm migration involved in gastrulation

Network	GO ID	GO description
Muscle	GO:0055001	muscle cell development
	GO:0042692	muscle cell differentiation
	GO:0007517	muscle organ development
	GO:0055002	striated muscle cell
	GO:0030017	sarcomere
	GO:0030016	myofibril
	GO:0043292	contractile fiber part
	GO:0043292	contractile fiber
	GO:0061061	muscle structure development
	GO:0046716	muscle cell cellular homeostasis
	GO:0006936	muscle contraction
	GO:0042692	muscle cell differentiation
	GO:0055002	striated muscle cell development
	GO:0006941	striated muscle contraction

Table S19. GO terms IDs used for the motif-associated GO term networks.

DNA libraries used for genome assembly

Species	DNA or RNA	Developmental stage	Library type	Paired or single end reads	Read length (bp)	Sequencer	Insert length (bp)	No. of reads (Million)
S. carpocapsae	DNA	IJ	regular	paired	75	Illumina Genome Analyzer IIx	400	85.76
S. carpocapsae	DNA	IJ	regular	paired	100	Illumina Genome Analyzer IIx	350	103.09
S. carpocapsae	DNA	IJ	jumping	paired	100	Illumina HiSeq2000	1800	131.59
S. feltiae	DNA	IJ	regular	paired	75	Illumina Genome Analyzer IIx	500	80.41
S. feltiae	DNA	IJ	regular	paired	100	Illumina HiSeq2000	450	75.94
S. glaseri	DNA	IJ	regular	paired	75	Illumina Genome Analyzer IIx	400	78.51
S. monticolum	DNA	IJ	regular	paired	75	Illumina Genome Analyzer IIx	390	108.61
S. scapterisci	DNA	IJ	regular	paired	75	Illumina Genome Analyzer IIx	390	101.64
S. scapterisci	DNA	IJ	regular	paired	100	Illumina HiSeq2000	340	97.54

Table S20. DNA library statistics for five *Steinernema* species.

RNA-seq libraries used for genome annotation

Species	DNA or RNA	Developmental stage	Stranded or unstranded	Paired or single end reads	Read length (bp)	Sequencer	Insert length (bp)	No. of reads (Million)
S. carpocapsae	RNA	embryo	unstranded	paired	75	Illumina Genome Analyzer IIx	200	54.94
S. carpocapsae	RNA	L1	unstranded	paired	75	Illumina Genome Analyzer IIx	200	65.96
S. carpocapsae	RNA	IJ	unstranded	paired	75	Illumina Genome Analyzer IIx	200	70.57
S. carpocapsae	RNA	adult	unstranded	paired	75	Illumina Genome Analyzer IIx	200	69.39
S. feltiae	RNA	IJ	unstranded	paired	75	Illumina Genome Analyzer IIx	200	87.99
S. glaseri	RNA	IJ	unstranded	paired	75	Illumina Genome Analyzer IIx	200	80.00
S. monticolum	RNA	IJ	unstranded	paired	75	Illumina Genome Analyzer IIx	200	95.74
S. scapterisci	RNA	IJ	unstranded	paired	75	Illumina Genome Analyzer IIx	200	83.83

Table S21. RNA library statistics for genome annotation.

	carpocapsae	*scapterisci*	*feltiae*	*glaseri*	*monticolum*
Augustus genes	28,313	31,378	33,459	34,143	36,007
Augustus transcripts	31,944	33,149	36,434	37,119	38,381
Additional novel Cufflinks genes	508	693	745	744	1,090
Additional novel Cufflinks transcripts	6,546	5,818	7,580	8,779	6,830

Table S22. Numbers of genes and transcripts that were predicted by Augustus for all *Steinernema* species, and numbers of novel genes and transcripts that were discovered with RNA-seq data sets using Cufflinks. Four RNA-seq data sets (embryo, L1, IJ, and adult) were used for novel gene and transcript finding for *S. carpocapsae*, while only an IJ-stage RNA-seq data set was using for all the other species.
