# Supplementary figures and images for: Comparative genomics of Steinernema reveals deeply conserved gene regulatory networks
Source: Genome Biol. 2015 Sep 21;16:200. doi: 10.1186/s13059-015-0746-6 (PMC4578762; doi:10.1186/s13059-015-0746-6)

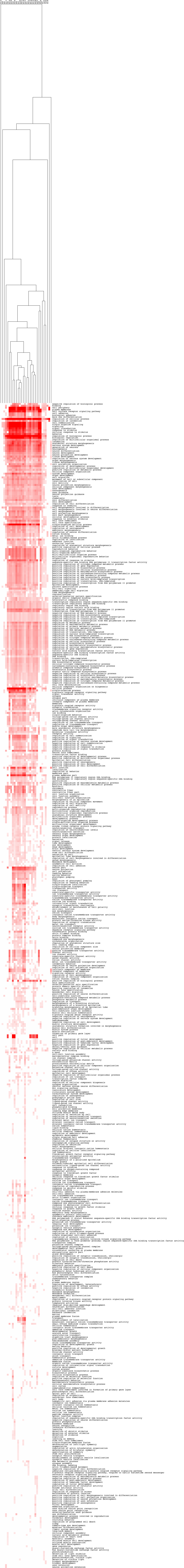

Supplement: Additional file 9: — S. carpocapsae and C. elegans predicted regulatory motifs GO term enrichments. Thirty significant, non-redundant motifs and the 619 GO terms they are enriched in for both S. carpocapsae and C. elegans. The heat map shows the –log10(p-value) for each motif-associated GO term. (PNG 1053 kb) [file 13059_2015_746_MOESM9_ESM.png]
